# Design Guidelines for Anode Modifications to Eliminate Soft Short Circuits in Anode‐Free Na Batteries Under High Current Density With Large‐Capacity Plating, Demonstrated via Initiated Chemical Vapor Deposition (iCVD)

**DOI:** 10.1002/anie.9967811

**Published:** 2026-06-07

**Authors:** Yeongjun Oh, Yuxuan Zhang, Jinwook Baek, Minyoung Kim, Jeongmin Park, Myung Gyu Lee, Chung Soo Kim, Sunghwan Lee

**Affiliations:** ^1^ School of Engineering Technology Purdue University West Lafayette Indiana USA; ^2^ Analysis and Standards Center Korea Institute of Ceramic Engineering and Technology (KICET) Jinju‐si Gyeongsangnam‐do Republic of Korea

**Keywords:** anode‐free, initiated chemical vapor deposition (iCVD), interphase design strategies, Na metal anode, soft‐short circuit

## Abstract

Soft short circuits, an underrecognized failure mode, are especially critical in anode‐free sodium systems, where limited Na inventory makes cells highly vulnerable to irreversible Na loss. However, clear interphase‐design principles for suppressing soft shorting remain lacking because its interfacial origin and governing descriptors are still poorly understood. Herein, we propose a mechanistically guided interphase design strategy for soft‐short‐free Na metal anodes under practical conditions. An ultrathin (20 nm) poly(1H,1H,7H‐dodecafluoroheptyl acrylate) (pDFHA) layer was conformally deposited on an Al current collector via a solvent‐free vapor‐phase polymerization process. Its minimal thickness and intrinsic ionic conductivity enabled favorable Na^+^ migration kinetics by shortening interfacial transport distance. Upon initial Na plating, interfacial conversion generated a ∼4 nm NaF‐rich inorganic domain, forming an integrated organic‐inorganic hybrid interphase. Finite element analysis showed that the laterally uniform interphase homogenized Na^+^ flux and mitigated localized current amplification, thereby markedly reducing soft short initiation. Meanwhile, the hybrid architecture combined mechanical strength with the ability to accommodate substantial volume fluctuation. As a result, symmetric cells with pDFHA‐modified electrodes cycled stably for over 2000 h without soft shorting. Anode‐free full cells paired with Na_3_V_2_(PO_4_)_3_ cathodes delivered 90.1 mAh g^−1^ at 1C and retained 90.8% capacity after 200 cycles, demonstrating practical robustness.

## Graphical abstract

This work establishes a laterally homogeneous organic–inorganic hybrid interphase for soft‐short‐free anode‐free sodium metal batteries under extended cycling at high current density and high plating capacity. In contrast to conventional strategies that evolve into laterally heterogeneous interphases and induce localized Na filament growth, the pDFHA‐derived lateral homogeneous interphase homogenizes interfacial Na^+^ flux and effectively suppresses soft‐short‐circuit initiation.

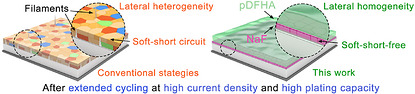

## 1 Introduction

**FIGURE 1:**
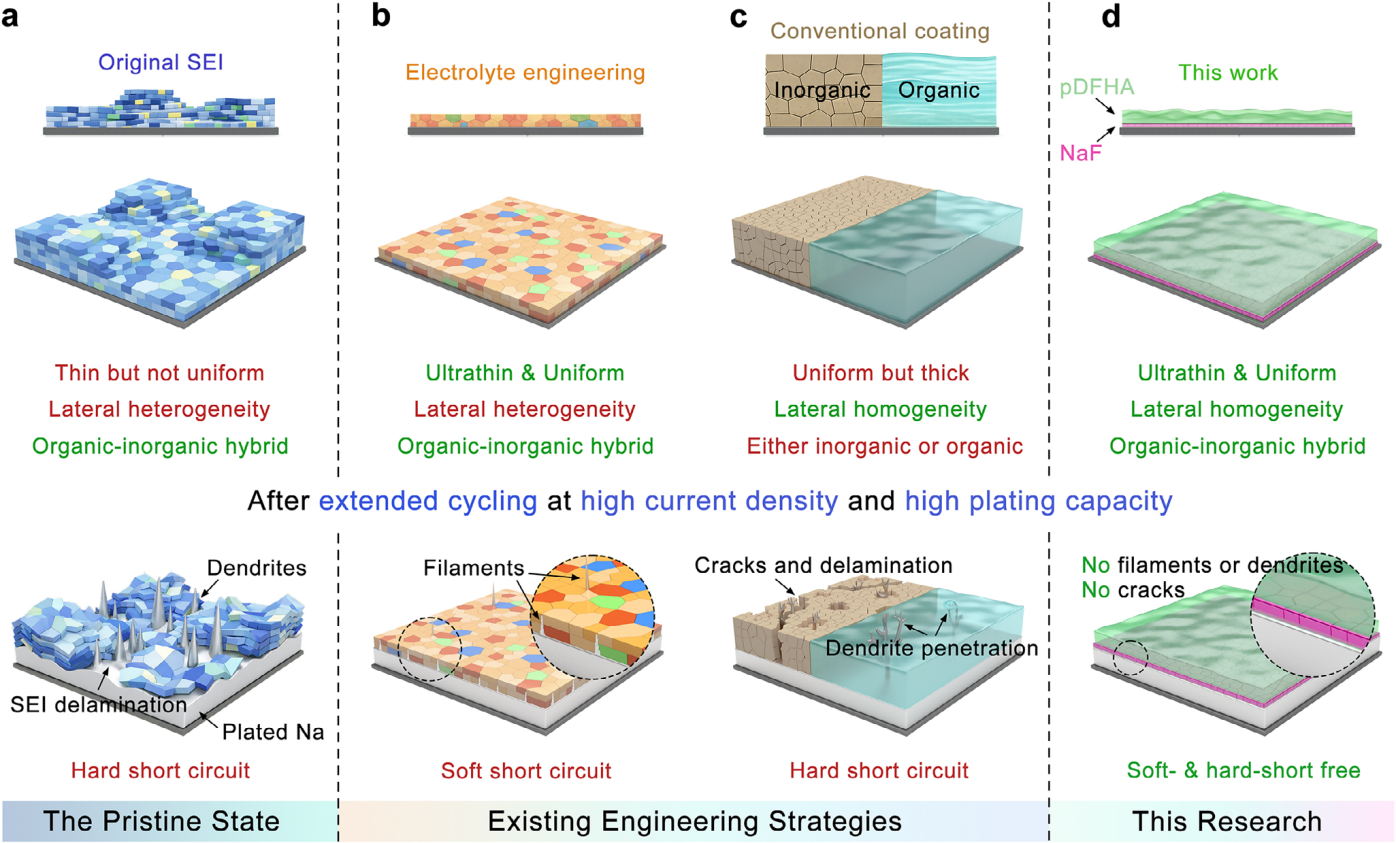
Schematic illustration of representative interphase design scenarios and their corresponding Na plating behaviors in anode‐free sodium systems: (a) native SEI, (b) an electrolyte‐engineering‐derived interphase, (c) an inorganic or organic artificial interphase, and (d) the interphase developed in this work.

Anode‐free full‐cell configurations, which use a current collector instead of excess Na metal at the anode side, have emerged as an effective strategy to maximize cell‐level energy density by minimizing inactive mass [1, 2, 3, 4]. However, their practical development is severely hindered by the poor reversibility of Na plating/stripping. The reductive decomposition of the electrolyte on deposited Na forms a solid electrolyte interphase (SEI), which tends to crack during repeated cycling because of large volume fluctuations [5, 6, 7, 8]. The resulting interfacial instability induces nonuniform Na deposition and dendrite growth, accelerates electrolyte consumption, lowers Coulombic efficiency (CE), and rapidly depletes the limited Na inventory in anode‐free systems, leading to severe capacity decay, short cycle life, and safety concerns, as shown in Figure 1a [1, 6, 9, 10].

To mitigate these challenges, electrolyte engineering has been widely adopted to stabilize Na deposition by regulating the formation of the SEI. Due to the self‐limiting nature of these reactions, the resulting SEI typically exhibits a nanoscale thickness, as evidenced by cryogenic microscopy revealing dimensions of ∼30 nm in fluoroethylene carbonate (FEC)‐containing carbonate electrolytes and ∼25–30 nm in optimized ether‐based systems, which is critical for facilitating fast Na^+^ transport kinetics by minimizing interfacial impedance and shortening diffusion pathways [5, 6, 11].

Despite these advances, electrolyte‐derived SEI layers are generally compositionally heterogeneous in the lateral direction due to the intrinsically stochastic nature of electrolyte decomposition and the spatially non‐uniform interfacial reaction environment [6, 12]. Although such lateral heterogeneity may not immediately trigger catastrophic hard short circuits, it can induce localized Na^+^ flux amplification at specific interfacial sites, especially under long cycling time with high current densities towards practical applications, thereby promoting localized nucleation and directional growth of plated Na [9, 12]. Specifically, once a localized Na^+^ flux reaches the metal anode surface, it can evolve into a thin metallic Na filament that preferentially propagates along gaps and voids within the SEI. Because such a filament is much thinner than a fully developed Na dendrite, its penetration through the separator and partial bridging of the cathode and anode does not necessarily result in an immediate hard short circuit, in which ionic charge transfer through the electrolyte is essentially disabled, and the cell response becomes dominated solely by an ohmic conduction pathway. Instead, the cell may enter a soft short‐circuit state, where charge transfer proceeds through the combined contributions of ionic transport and electronic conduction [9, 13]. Accordingly, the most informative electrochemical indicators of soft shorting are the coexistence of residual mass‐transport polarization with an abnormally enhanced Ohmic contribution, together with a pronounced reduction in the apparent cell impedance arising from the emergence of a parallel electronic leakage pathway. Because certain soft shorts may self‐heal during subsequent stripping/plating cycles (e.g., via filament dissolution), this phenomenon has historically received less attention. However, recent studies indicate that such self‐healing occurs only under limited and not yet fully understood conditions. Moreover, even when a soft short circuit does not immediately progress into a hard short, the partial bridging of the electrodes by a metallic filament can still enable parasitic electron leakage, leading to continuous self‐discharge and loss of stored energy. In addition, the electronically conductive filament creates localized electrochemically active sites and abnormal current concentration, which can continuously drive electrolyte reduction at the filament surface and nearby interfacial regions. As a result, soft short circuits can accelerate electrolyte decomposition, intensify interfacial degradation, and promote the progressive deterioration of cell stability [13, 14, 15]. Over extended cycling, these effects may result in capacity fading, CE decay, and ultimately irreversible cell failure, as indicated in Figure 1b [13, 14, 15, 16].

Although soft short behavior was not examined in earlier studies, some artificial coating approaches might have incidentally alleviated soft short formation during the initial cycles [17]. However, inorganic artificial coatings typically perform effectively only under low current densities and limited plating capacities, where volume variation during Na deposition remains moderate, a condition that deviates from practical operating environments [18, 19]. In contrast, when the cell is subjected to higher current densities and larger plating amounts, substantial volumetric expansion of Na generates significant interfacial stresses that rigid inorganic coatings struggle to accommodate, leading to mechanical fracture and failure, as demonstrated in Figure 1c [17, 18, 19, 20], thereby eventually causing short circuits. Purely organic protective layers, owing to their intrinsic resilience, are generally capable of accommodating interfacial volume fluctuations. Nevertheless, their insufficient mechanical strength limits their ability to effectively resist metal dendrite penetration, as illustrated in Figure 1c. In addition, artificial coatings that rely on solution‐based techniques generally require coating materials to be soluble in solvents and typically yield micrometer‐thick layers, which not only restrict material selection but also prolong the Na^+^ migration pathway, thereby compromising interfacial kinetics.

Based on the above considerations, this study aims to develop a soft‐short‐free Na metal anode that enables favorable Na^+^ transport and practically durable operation under high current densities and large plating capacities. To achieve this goal, the interfacial layer must meet several stringent requirements simultaneously. Specifically, we hypothesize that: (1) soft short behavior can be suppressed by establishing a laterally compositionally homogeneous interphase to eliminate localized Na^+^ flux heterogeneity; (2) efficient Na^+^ migration can be preserved through an ultrathin interphase with a thickness of approximately 20–70 nm, comparable to that of the native SEI; and (3) stable operation under practically relevant plating amount can be achieved through an organic‐inorganic hybrid architecture that combines mechanical strength with the ability to accommodate interfacial deformation.

These hypotheses were formulated on the basis of several well‐established considerations. First, localized Na^+^ flux concentration induced by compositional random interphase can lead to preferential Na accumulation, whereas a laterally compositionally homogeneous interphase may eliminate horizontal variations in Na^+^ flux and thereby suppress the formation of localized Na filaments [12, 19]. Second, the Na^+^ migration length across the interfacial layer is a key factor governing Na^+^ plating kinetics; an ultrathin interphase reduces the Na^+^ transport distance to only tens of nanometers, thereby promoting rapid interfacial ion transport [6, 11]. Third, an organic–inorganic hybrid interphase may simultaneously provide sufficient mechanical strength to suppress dendrite penetration and adequate resilience to accommodate the large volume fluctuations associated with Na^+^ plating under high current densities and large capacities.

To realize such an interfacial layer, an appropriate fabrication method must be carefully selected to simultaneously satisfy the above requirements. Vapor‐phase deposition is particularly more attractive in this regard because it enables the formation of ultrathin and uniform coatings compared to the solution‐based strategies. However, conventional vapor‐phase techniques, such as sputtering and atomic layer deposition, are primarily limited to inorganic materials and therefore cannot readily generate the organic component required for constructing an organic‐inorganic hybrid [21]. By contrast, polymer‐based coating strategies offer a distinct advantage: when a rationally selected polymer is employed, its functional groups can spontaneously react with freshly plated Na to form inorganic species in situ, while the unreacted polymer matrix remains as the organic component. In this way, an integrated organic‐inorganic hybrid interphase can be established without the need for multiple deposition processes.

In principle, vapor‐phase polymerization can proceed through either step‐growth or chain‐growth mechanisms [22]. However, step‐growth routes often produce conjugated polymer backbones with delocalized pi‐electron structures, resulting in undesirably high electronic conductivity for anode protection, where the interfacial layer should remain electronically insulating while allowing Na^+^ transport [23, 24].

Accordingly, a vapor‐phase free‐radical chain‐growth polymerization route, namely initiated chemical vapor deposition (iCVD), is particularly suitable for constructing the desired interfacial layer.

To rationalize the choice of polymer chemistry, we further screened candidate materials from the perspective of the inorganic species expected to form upon reaction with plated Na, thereby defining the functional groups required in the precursor monomer. Among the possible Na‐containing inorganic products, such as Na_2_O, NaF, and Na_2_CO_3_, NaF is particularly attractive because of its high chemical stability, wide electrochemical stability window, and favorable formation tendency. On this basis, F‐rich polymers were identified as especially promising candidates for interphase design. For this reason, poly(1H,1H,7H‐dodecafluoroheptyl acrylate) (pDFHA, 12 F per molecule) was selected as the precursor coating material, as its high density of ‐CF_2_‐containing side chains provides abundant fluorinated functional groups for interfacial conversion of plated Na.

To validate our hypothesis, a uniform and conformal pDFHA coating with a thickness of approximately 20 nm was deposited on the Al current collector via iCVD technique. Subsequent x‐ray photoelectron spectroscopy (XPS) depth profiling and cross‐sectional transmission electron microscopy (TEM) characterization confirm that during the initial Na deposition process, interfacial reactions between Na and the fluorinated moieties generate an approximately 4 nm NaF‐rich inorganic inner layer, while the remaining unreacted polymer constitutes the outer organic framework. This results in a vertically integrated organic–inorganic hybrid interphase composed of a NaF‐rich bottom layer coupled with a polymer‐rich top layer, as shown in Figure 1d. Finite element analysis (FEA) further reveals that even when an interphase maintains uniform thickness, lateral compositional heterogeneity can induce localized Na^+^ flux amplification, which serves as a precursor to soft short circuit formation. In contrast, the pDFHA‐derived interphase, characterized by horizontally uniform chemical composition, effectively redistributes the Na^+^ flux into a laterally homogeneous pattern, thereby mitigating local current concentration and suppressing Na filament initiation (i.e., soft short).

Consistent with this mechanistic understanding, symmetric cells employing pristine electrodes exhibit pronounced voltage fluctuations characteristic of soft shorting, during which the mass‐transport contribution is markedly reduced relative to the ohmic contribution, accompanied by a decrease in impedance, suggesting a soft short circuit behavior. This process ultimately evolves into hard short failure, where the cell response becomes dominated by the ohmic component. In contrast, symmetric cells based on pDFHA‐modified electrodes maintain stable cycling for over 2000 h at 1 mA cm^−2^, where the mass‐transport contribution remains evident, and the impedance increases only slightly during cycling, indicating that no soft short circuit occurs throughout operation. Moreover, the symmetric cells with the pDFHA@Al electrode even exhibit stable cycling under 2 mA cm^−2^ for 1000 h and under high current density of 5 mA cm^−2^ for 500 h without soft short circuit.

To evaluate the practical applicability of the engineered interphase, full cells were assembled using Na_3_V_2_(PO_4_)_3_ (NVP) cathodes with varying areal loadings to simulate different Na plating capacities. Importantly, the NVP cathode with a mass loading of 5.6 mg cm^−2^ was paired with a pDFHA@Al electrode, which corresponds to a negative to positive (N:P) ratio of 0, and delivered a specific capacity of 90.1 mAh g^−1^ and maintained its 90.8% of its original capacity after 200 cycles at 1 C.

This work not only provides a practical strategy for realizing soft‐short‐free operation in anode‐free Na metal batteries but also offers a mechanistic framework for interphase design toward stable alkali metal anodes under practically demanding conditions.

## 2 Results and Discussion

**FIGURE 2:**
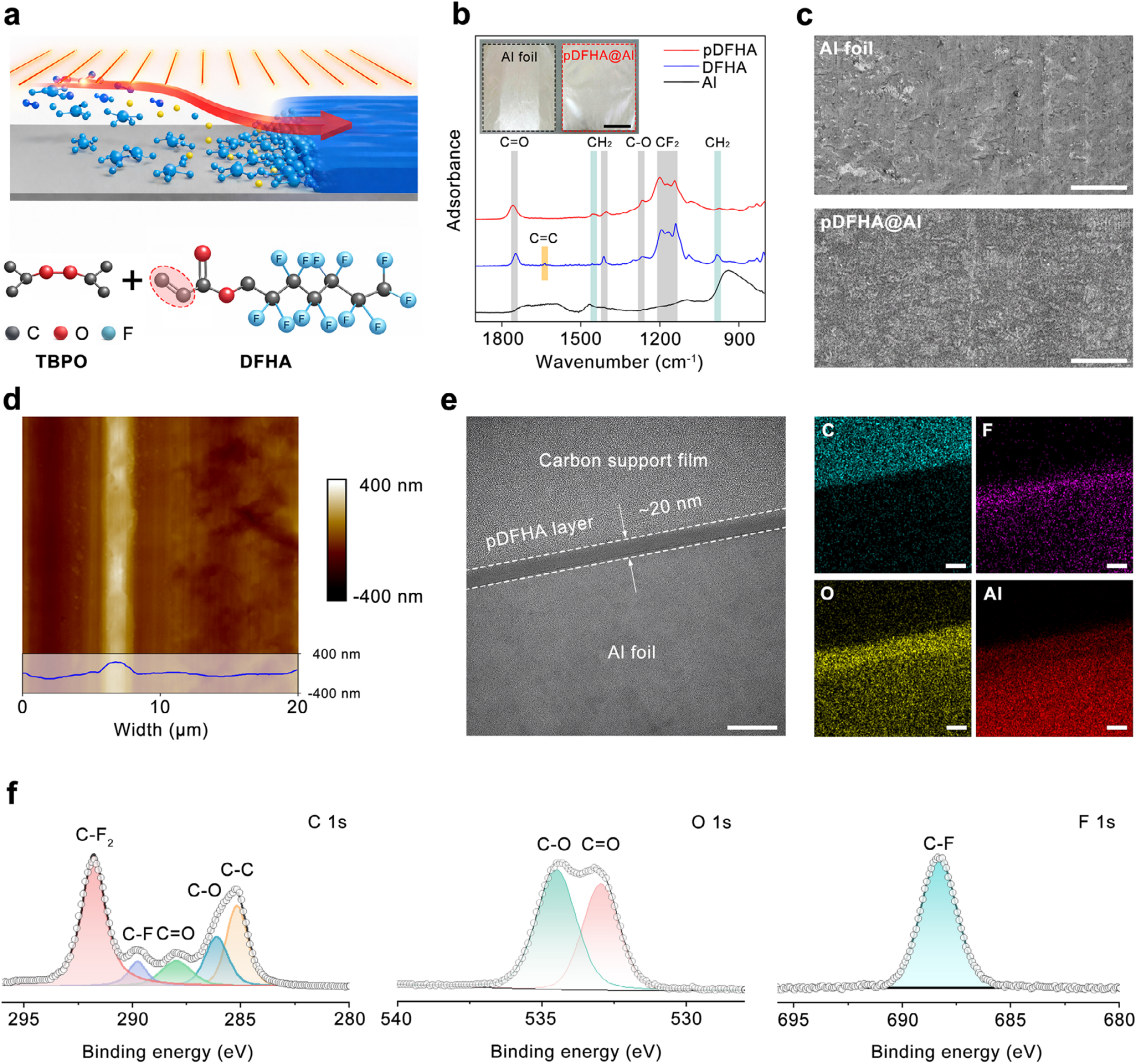
Design, fabrication, and initial verification of iCVD‐grown pDFHA thin films on an Al current collector. (a) Schematic illustration of the iCVD polymerization of DFHA on Al using TBPO as the radical initiator. (b) Optical photographs (inset, scale bar: 10 cm) of bare Al and pDFHA@Al and FT‐IR spectra of bare Al, liquid DFHA monomer, and pDFHA@Al. (c) Top‐view SEM image of bare Al and pDFHA@Al (scale bars: 20 µm). (d) Two‐dimensional AFM height map of pDFHA@Al with the corresponding line profile. (e) Cross‐sectional TEM image of as‐manufactured pDFHA@Al (scale bar: 50 nm) and the corresponding EDX elemental maps (scale bar: 20 nm). (f) High‐resolution XPS spectra of pDFHA@Al in the C 1s, O 1s, and F 1s regions.

Figure 2a schematically illustrates the iCVD process used to form a fluorine‐rich pDFHA thin film on the Al current collector. In this all‐dry polymerization, vapors of 1H,1H,7H‐dodecafluoroheptyl acrylate (DFHA, H_2_C═CHCO_2_CH_2_(CF_2_)_5_CHF_2_, containing 12 fluorine atoms per monomer unit) monomer and an initiator, tert‐butyl peroxide (TBPO, (CH_3_)_3_COOC(CH_3_)_3_) in this case, are introduced into a low‐pressure reaction chamber and flow past an array of resistively heated filaments at the top of the reactor. Near these filaments, the initiator thermally decomposes to generate free radicals, while the DFHA molecules remain intact and adsorb on the temperature‐controlled Al foil substrate at the bottom. Radical species that reach the substrate surface initiate free‐radical chain polymerization of DFHA, in which double bonds (marked in Figure 2a as an oval) are broken and converted into polymer chains, enabling direct growth of a pDFHA polymer film on the Al current collector [25]. Fourier‐transform infrared (FT‐IR) spectra of bare Al, liquid DFHA monomer, and pDFHA@Al were collected to verify the DFHA‐to‐pDFHA conversion and the retention of fluorinated ester functionalities after iCVD deposition (Figure 2b). The bare Al spectrum displays only a single broad feature around ∼950 cm^−1^, which can be attributed to native surface oxide or hydroxyl species. The liquid DFHA monomer exhibits vinyl‐related absorptions at ∼1650 cm^−1^ (acrylate C═C stretching), ∼980 cm^−1^ (out‐of‐plane C‐H wagging of the terminal ═CH_2_ group), and ∼1420 cm^−1^ (═CH_2_ bending), together with fluorinated ester signatures including the ester C═O band near ∼1730 cm^−1^, the C─O/C─O─C band at ∼1268 cm^−1^, and CF_x_ bands at ∼1200, 1164, and 1134 cm^−1^ assigned to CF_2_H, CF_2_, and CH_2_CF_2_ stretching modes within the dodecafluoroheptyl side chain. Compared with DFHA, the most prominent change after iCVD deposition is the suppression of vinyl‐related absorptions and the emergence of saturated backbone signatures. In the pDFHA@Al spectrum, the ∼1650 cm^−1^ band disappears within the detection limit, the 980 cm^−1^ band becomes markedly weaker, and the ∼1420 cm^−1^ feature is replaced by bands at ∼1400 and ∼1460 cm^−1^ characteristic of saturated ─CH_2_─ deformation modes in polymer backbones and side chains. These changes demonstrate that the terminal vinyl groups are largely consumed and a saturated polymer backbone is formed on the Al surface during iCVD polymerization. Moreover, the inset of Figure 2b shows an optical photograph of a large‐format pDFHA@Al foil (25 cm × 30 cm), where the coated foil retains a metallic appearance with a macroscopically uniform surface devoid of obvious large‐scale defects or discoloration, confirming the manufacturability of the iCVD process for large‐area production.

The surface morphology of the Al foil before and after pDFHA coating was examined and compared using top‐view scanning electron microscopy (SEM) (Figure 2c). As a baseline, the SEM image of bare Al (Figure 2c, top) displays elongated rolling marks and sharply defined grooves, typical of mechanically rolled Al. Comparatively, the pDFHA@Al surface (Figure 2c, bottom) retains a similar striated texture with slightly rounded groove edges, yet is free of contaminant particles, cracks, or bright metallic patches corresponding to exposed Al. Similar surface morphologies have been reported for iCVD‐grown polymer films, where the deposited layer follows the underlying surface geometry without introducing additional surface non‐uniformity or defects, demonstrating the excellent conformal coating capability of iCVD [26, 27]. Collectively, these observations suggest that the iCVD‐derived pDFHA layer preserves the existing foil texture while forming a laterally continuous polymer film at the micrometer scale.

To further quantify this surface uniformity, atomic force microscopy (AFM) was employed to probe the nanoscale topography of bare and pDFHA‐coated Al surfaces in a 20 µm × 20 µm area (Figure S1 and Figure 2d). The bare Al foil (Figure S1) exhibits pronounced ridge‐ and valley‐like features with a relatively rough height profile that follows the rolling marks, reflecting the mechanically processed texture of the current collector, yielding a root‐mean‐square roughness (RMS) of 83.5 nm. In contrast, the two‐dimensional AFM height map and corresponding line profile of pDFHA@Al (Figure 2d) reveal a similar characteristic of the rolling marks in the bare Al foil and an approximate RMS (76.1 nm), suggesting the pDFHA polymer coating conforms to the Al substrate rather than introducing additional roughness.

The cross‐sectional TEM image reveals a continuous layer with a thickness of ∼20 nm, which can be assigned to the pDFHA coating, indicating the uniformity and ultrathin thickness of the film. In addition, the polymer layer is intimately contacted to the Al foil with no interfacial voids, cracks, or delaminated regions observed. Energy‐dispersive x‐ray spectroscopy (EDX) elemental mapping shows that the carbon signal is mainly localized in the upper‐left region, originating from the carbon support film of the TEM grid.

In contrast, O and F, the characteristic elements originating from the DFHA monomer unit, are uniformly distributed within the pDFHA coating region, suggesting a homogeneous composition of the deposited pDFHA layer. A strong Al signal is predominantly detected in the lower region, further confirming that the pDFHA coating is homogeneously formed in situ on the surface of the Al foil substrate.

XPS measurements were carried out to gain deeper insight into the surface chemistry of the as‐deposited pDFHA coating layer grown on the Al current collector. The survey spectrum of pDFHA@Al exhibits major elements of C 1s, F 1s, and O 1s signals, while no discernible Al 2p feature is observed in a high‐resolution Al 2p scan (Figure S2), suggesting the XPS‐probed near‐surface region is governed by the polymer overlayer rather than the metallic substrate. To elucidate the bonding environments of ester‐ and fluorinated moieties in pDFHA, high‐resolution XPS spectra were collected in the C 1s, O 1s, and F 1s regions (Figure 2f). In the C 1s region, the component centered at ∼284.8–285.2 eV can be assigned to aliphatic C─C/C─H, the peak at ∼286.3 eV corresponds to C─O, and the peak at ∼288.2 eV is attributed to carbonyl C═O environments [28, 29]. Additional higher‐binding‐energy contributions at ∼289–290 eV and ∼291–292 eV are assigned to C─F and CF_2_ units within the dodecafluoroheptyl side chains of DFHA‐derived segments. The O 1s envelope can be described by two components at ∼532–533 eV and ∼534–535 eV, which arise from two oxygen environments in the ester moiety, namely alkoxy oxygen (C─O) and carbonyl oxygen (C═O) in the DFHA‐derived backbone. The F 1s spectrum is dominated by a single peak centered at ∼688–689 eV, characteristic of fluorine in covalent C─F bonds within the fluorinated side chains. These XPS results validate that the DFHA‐derived ester backbone and dodecafluoroheptyl side chains remain chemically intact and that the as‐deposited pDFHA presents a fluorine‐rich surface.

The effect of pDFHA coating on the kinetics behavior of Na^+^ transmission and plating was investigated through multiple electrochemical methods. To quantify the ionic conductivity (*σ*_i,pDFHA_) and electronic conductivity (*σ*_e,pDFHA_) of the iCVD‐grown pDFHA coating, measurements were performed. As indicated in Figures S3 and S4, the *σ*_i,pDFHA_ and *σ*_e,pDFHA_ were calculated as 1.44 × 10^−6^ S cm^−1^ and 1.72 × 10^−10^ S cm^−1^, respectively, which indicates that the pDFHA coating effectively blocks electron transport while remaining relatively permeable to ion transport.

Chronoamperometry (CA) measurements were conducted on bare Al and pDFHA@Al electrodes at a constant potential of −0.6 V to evaluate Na nucleation and subsequent early‐stage growth kinetics. For metal electrodeposition, the transient response can generally be interpreted in terms of the initial formation of stable nuclei followed by their subsequent enlargement over time.

As shown in Figure S5, the resulting current transients display the characteristic evolution of a nucleation process. The current initially increases as the electrochemically active area expands through the formation and growth of Na nuclei. The current then reaches a maximum value (*i*_m_) at a specific time (*t*_m_) and eventually declines as the mass transfer shifts from hemispherical to linear diffusion. From these transient responses, the pDFHA@Al electrode yields a higher *i*_m_ of 45.4 mA cm^−2^ and a shorter *t*_m_ of 7.3 s compared to the 33.8 mA cm^−2^ and 15.8 s observed for bare Al. This accelerated response indicates faster nucleation and growth kinetics that drive an earlier transition to the linear diffusion regime, suggesting that the pDFHA coating effectively lowers the energy barrier for Na nucleus formation to facilitate highly efficient Na plating. To further elucidate the nucleation mechanism, the transient responses were analyzed using the Scharifker–Hills (SH) model. According to the SH theory, diffusion‐controlled 3D nucleation and growth are categorized into two distinct models, comprising instantaneous nucleation and progressive nucleation. Specifically, instantaneous nucleation enables the near‐simultaneous activation of surface nucleation sites, which distributes Na plating more uniformly at the initial stage, thereby minimizing the dominance of isolated hot spots and promoting spatially uniform Na growth. By contrast, progressive nucleation reflects temporally continuous and spatially nonuniform nucleus generation, under which earlier formed or kinetically favored nuclei tend to capture more Na^+^ flux and grow preferentially. This disparity in local growth accelerates surface roughening, amplifies current localization, and increases the probability of filamentary Na formation. The corresponding normalized dimensionless equations are given in Equations (1) and (2) [30]:

For instantaneous nucleation,

(1) $$\begin{array}{c} \;{\left(\frac{I}{I_{m}}\right)}^{2}=\frac{1.9542}{\frac{t}{t_{m}}}\;{\left\{1-\exp\left[-1.2564\left(\frac{t}{t_{m}}\right)\right]\right\}}^{2} \end{array}$$

For progressive nucleation,

(2) $$\begin{array}{c} \;{\left(\frac{I}{I_{m}}\right)}^{2}=\frac{1.2254}{\frac{t}{t_{m}}}\;{\left\{1-\exp\left[-2.3367{\left(\frac{t}{t_{m}}\right)}^{2}\right]\right\}}^{2} \end{array}$$

**FIGURE 3:**
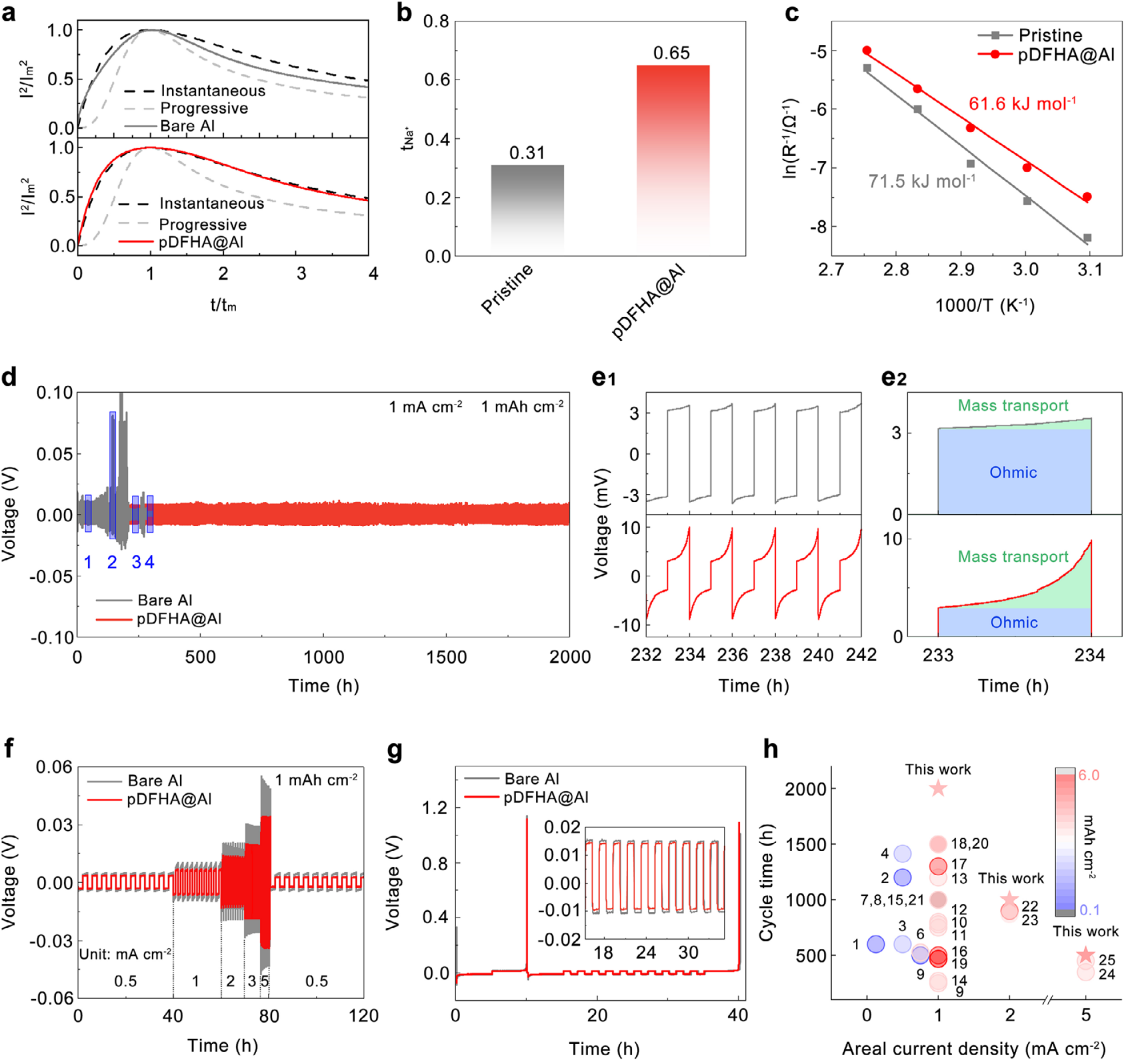
Electrochemical kinetics and interfacial stability analysis of the electrodes. (a) Dimensionless current‐time transients derived from chronoamperometry for bare Al and pDFHA@Al electrodes, compared with the Scharifker–Hills theoretical models for instantaneous and progressive nucleation. (b) Calculated Na^+^ transference numbers for bare Al and pDFHA@Al electrodes derived from potentiostatic polarization and EIS. (c) Arrhenius plots of inverse charge‐transfer resistance versus inverse temperature to determine the activation energy for Na^+^ desolvation. (d) Long‐term galvanostatic cycling stability of symmetric cells using bare Al and pDFHA@Al electrodes at 1 mA cm^−2^ with a fixed capacity of 1 mAh cm^−2^. (e1) Enlarged voltage profiles during extended cycling and (e2) corresponding overpotential contributions decoupled into Ohmic and mass transport polarizations for both cells. (f) Rate performance of the symmetric cells at various current densities from 0.5 to 5 mA cm^−2^. (g) Average Coulombic efficiency measurements using the Aurbach‐type partial‐stripping protocol. (h) A comparison of this work with representative anode‐free Na interface‐modification strategies [1, 18, 36, 37, 38, 39, 40, 41, 42, 43, 44, 45, 46, 47, 48, 49, 50, 51, 52, 53, 54, 55, 56].

Here, *i* and *t* denote current density and time, respectively. Figure 3a compares the normalized experimental curves derived from Figure S5 with the theoretical curves calculated from Equations (1) and (2). While pDFHA@Al (Figure 3a, lower) consistently follows the instantaneous model, the bare Al electrode (Figure 3a, upper) exhibits nucleation behavior that deviates substantially from the instantaneous model and rather shifts toward the progressive model. Together with the higher *i*_m_ and shorter *t*_m_ values, this result further substantiates the interpretation that pDFHA promotes more favorable Na nucleation and subsequent growth kinetics on the Al surface.

To quantify the difference in Na^+^ transference number between cells with the bare Al and pDFHA@Al, direct current polarization coupled with impedance measurement was performed (Figure S6). The Na^+^ transference number (*t*_Na_^+^) was calculated using the Bruce–Vincent equation [31]:

(3) $$\begin{array}{c} \;{t_{Na}}^{+}=\frac{I_{ss}\left(\Delta V-I_{0}R_{0}\right)}{I_{0}\left(\Delta V-I_{ss}R_{ss}\right)}\;, \end{array}$$

where Δ*V* is the applied polarization voltage (10 mV), I_0_ and *I*_ss_ are the initial and steady‐state currents obtained from the CA profiles, and *R*_0_ and *R*_ss_ are defined as the total interfacial resistances extracted from the Nyquist plots before and after polarization, respectively. As summarized in Figure 3b, the pDFHA@Al electrode yields a high *t*_Na+_ of approximately 0.65, which is more than double that of the bare Al (∼0.31). This higher cation transference number reduces interfacial concentration gradients and delays the onset of Sand's time, thereby helping to suppress dendrite initiation during metal deposition.

To evaluate the effect of pDFHA‐derived interphase on activation energy for the Na^+^ desolvation process, temperature‐dependent in situ electrochemical impedance spectroscopy (EIS) was performed from 25 to 65°C as indicated in Figure S7. The charge‐transfer resistance (*R*_ct_) obtained from these spectra was used to construct Arrhenius plots in Figure 3c, where the linear fit of ln(*R*_ct_^−1^) versus 1000/*T* allows for the determination of the activation energy associated with desolvation (*E*_a_) [32]:

(4) $$\begin{array}{c} l\;n\left(R_{ct}^{-1}\right)=-lnR_{0}-\frac{E_{a}}{1000R}\left(\frac{1000}{T}\right), \end{array}$$

where *R*_0_ represents the pre‐exponential constant obtained from Arrhenius fitting, *R* is the gas constant and *T* is the absolute temperature. According to this relationship, the slope of the linear fit corresponds to −*E*_a_/*R*. The pristine Al electrode exhibits a steeper slope, corresponding to a high activation energy of approximately 71.5 kJ mol^−1^. In contrast, the pDFHA@Al electrode shows a reduced slope with a lower activation energy of approximately 61.6 kJ mol^−1^, indicating a lower kinetic barrier for interfacial Na^+^ desolvation at the interface of the electrode and electrolyte.

The effect of the pDFHA coating on the electrochemical stability during the Na plating/stripping over prolonged operation was evaluated by symmetric cells. Long‐term galvanostatic cycling was then performed at a current density of 1 mA cm^−2^ with a fixed capacity of 1 mAh cm^−2^ (Figure 3d). The initial galvanostatic plating profiles show that pDFHA@Al exhibits significantly lower polarization than bare Al during the initial nucleation process (−17 mV for pDFHA@Al vs. −46 mV for bare Al) as indicated in Figure S8, which suggests the cell with the pDFHA@Al shows a lower nucleation energy barrier. Moreover, the cell with the pDFHA@Al electrode exhibited a smaller impedance value of 65 Ω compared with 127 Ω for bare Al, which further evidences the more favorable kinetics of pDFHA@Al (Figure S9). Cyclic voltammetry measurements further revealed distinct differences in the Na deposition/dissolution behavior between the two electrodes. As shown in Figure S10, pDFHA@Al exhibited higher current density during Na plating/stripping than bare Al, indicating more facile Na redox kinetics. The initial CE of the cell with pDFHA@Al (88.6%) electrode is markedly higher than that of the cell with the bare Al (68.5%) as presented in Figure S11. This difference indicates more efficient and reversible Na utilization during the initial cycle for the pDFHA@Al electrode, whereas the bare Al electrode suffers from lower reversibility due to Na consumption associated with native SEI formation.

In Figure 3d, the symmetric cell utilizing pDFHA@Al electrode maintains stable cycling for over 2000 h, whereas the cell with bare Al exhibits inferior stability and short‐circuits at approximately 285 h. In addition, the pDFHA@Al cell sustains a low and stable polarization of approximately 20 mV without pronounced growth during extended operation, indicating a kinetically stable interface. In contrast, the bare Al cell displays increasing polarization and erratic voltage fluctuations prior to failure.

To systematically evaluate the influence of the pDFHA@Al electrode on the short‐circuit behavior, four representative voltage segments (marked by blue rectangles) were extracted from the long‐term cycling profile and analyzed individually. As shown in Figure S12, both the pDFHA@Al‐based cell and the bare Al‐based cell exhibit similar electrochemical characteristics during Stage 1. The voltage profiles display an initial Ohmic polarization followed by a sloping behavior associated with ionic mass transport, which is the typical signature of a normally functioning symmetric cell in which the current is fully carried by ionic conduction. This indicates that neither cell experiences internal shorting at this stage.

A clear divergence emerges in Stage 2. As indicated in Figure S12, the cell employing the pDFHA@Al electrode maintains a stable voltage profile with characteristics identical to those observed in Stage 1. In contrast, the bare Al‐based cell begins to exhibit pronounced voltage spikes and irregular fluctuations. Such a fluctuating yet non‐collapsed voltage response, which appeared after 100 h, is fundamentally different from the transient fluctuations observed during the initial ∼10 h, which can still be associated with early‐stage SEI formation and interfacial stabilization. At this later stage, when a passivating interphase has already been established, such recurrent voltage oscillations are more reasonably ascribed to the onset of soft shorting. Specifically, the temporary voltage drops (as marked by the arrow in Figure S12) can be attributed to partial electronic bridging between the two electrodes by Na filaments, whereas the subsequent voltage recovery likely results from transient filament disconnection induced by Joule‐heating‐driven melting, collapse, or dissolution. The repeated alternation between these two states is therefore consistent with the dynamic un‐shorting/re‐shorting behavior widely recognized as a hallmark of early‐stage soft shorts [18, 33, 34].

The evolution becomes more evident in Stage 3 (Figure 3e). The pDFHA@Al cell continues to exhibit a typical plating/stripping voltage profile dominated by ionic transport as indicated in Figure 3e1. In contrast, the bare Al cell no longer shows pronounced voltage spikes; instead, its voltage curve becomes noticeably flatter with a significantly reduced slope. Detailed analysis of the voltage response in Figure 3e2 within the 233–234 h interval reveals that the pDFHA@Al cell still retains a substantial contribution from mass‐transport polarization, indicating that the current is predominantly governed by ionic transport processes, consistent with normal electrochemical operation. For the bare Al cell, however, the contribution from mass transport becomes minimal, and the voltage response is dominated by Ohmic polarization. This suggests that most of the charge transfer is now carried through electron conduction pathways, while only a small fraction still proceeds through ionic transport driven by concentration and electric‐field gradients. Because electronic conduction through metallic filaments is kinetically much faster than ionic migration, the required charge can be transferred rapidly, allowing the cell to reach the preset capacity limit within a shorter time. Furthermore, because the electron pathway bypasses the electrochemical reaction resistance, the potential difference between the two electrodes is not significantly elevated, resulting in a substantially lower apparent overpotential compared with the pDFHA@Al cell. Importantly, the presence of residual ionic transport indicates that electrochemical reactions are still partially occurring within the cell. Note that this situation differs fundamentally from a conventional hard short circuit, in which electrochemical processes are completely suppressed. Therefore, the bare Al cell in Stage 3 can be identified as operating under a soft short‐circuit condition, where ionic and electronic currents coexist simultaneously within the system.

Finally, in Stage 4, a clear transition to a hard short circuit is observed for the bare Al cell. As shown in Figure S12, the pDFHA@Al cell still maintains a plating/stripping voltage profile with noticeable mass‐transport polarization. In contrast, the voltage curve of the bare Al cell collapses into an almost perfectly horizontal line. Such behavior indicates that the cell response has become purely electronic, where the current is entirely carried by electronic conduction through a permanent internal connection between the electrodes. This is the characteristic electrochemical signature of a hard short circuit, confirming that a stable metallic filament has formed and fully bridged the two electrodes. In general, this evolution from soft short to hard short can be understood as a progressive process in which a transient and metallic filament becomes thicker, more stable, and more electronically dominant because of continued localized Na deposition. In addition, to further substantiate the voltage‐profile analysis, EIS was performed on a separate set of cells at the four representative stages discussed above. As shown in Figure S13, both cells exhibit typical semicircular impedance responses in Stage 1, consistent with normal interfacial charge‐transfer and ionic transport processes in a functioning symmetric cell. As cycling proceeds, the impedance evolution of the two cells becomes markedly different. The pDFHA@Al cell maintains a nearly unchanged impedance response from Stage 1 to Stage 4, confirming the persistent interfacial stability of the engineered interphase. In contrast, the bare Al cell first exhibits an enlarged impedance response in Stage 2, suggesting progressive interfacial degradation prior to failure, and then shows a pronounced collapse of the impedance spectrum in Stages 3 and 4. Such a transition is consistent with the emergence of an internal electronic conduction pathway associated with Na filament formation, corresponding to soft shorting in Stage 3 and eventual hard shorting in Stage 4. In particular, the coexistence of a markedly reduced mass‐transport contribution with a preserved residual impedance feature in the pristine cell indicates a soft‐short state, whereas the nearly complete loss of mass‐transport polarization together with the collapse of impedance confirms the transition to a hard short.

The rate performance of the symmetric cells using pDFHA@Al and bare Al electrodes was investigated at a fixed areal capacity of 1 mAh cm^−2^ (Figure 3f). As the current density was increased from 0.5 to 5 mA cm^−2^, the overpotential of both cells increased accordingly. Notably, the pDFHA@Al cell consistently maintained a lower overpotential with stable plating/stripping profiles across all current densities, specifically ∼4 mV at 0.5 mA cm^−2^ and ∼35 mV at 5 mA cm^−2^. In sharp contrast, the bare Al cell exhibited a significantly steeper polarization rise, reaching ∼8 mV at 0.5 mA cm^−2^ and exceeding 50 mV at 5 mA cm^−2^, which suggests the potential of the pDFHA@Al electrode utilized under practical fast charging conditions due to the more favorable interfacial Na plating kinetics. In addition, long‐term galvanostatic cycling was further conducted at constant current densities of 2 and 5 mA cm^−2^ (corresponding to 2 and 5 mAh cm^−2^ per cycle, respectively, Figures S14 and S15) to represent a practical operating scenario. The pDFHA@Al cell maintained stable plating/stripping profiles for over 1000 h at 2 mA cm^−2^ and over 500 h at 5 mA cm^−2^ without signs of dendritic shorting. Conversely, the pristine bare Al cell failed rapidly under these aggressive conditions, exhibiting erratic voltage fluctuations and short‐circuits within approximately 350 h at 2 mA cm^−2^ and roughly 180 h at 5 mA cm^−2^. To further assess the electrolyte compatibility of the pDFHA‐derived interphase, we additionally evaluated the plating/stripping stability of pristine and pDFHA‐modified electrodes in a more conventional carbonate‐based electrolyte under different testing conditions. As shown in Figure S16, the pDFHA‐derived interphase still maintains clearly improved cycling stability in the ester‐based electrolyte, further supporting its practical capability beyond the ether‐based system. These results demonstrate that the pDFHA‐modified electrode enables substantially improved interfacial stability to suppress the soft short circuit under high‐current‐density Na plating/stripping with extended operation time, even in the more challenging carbonate‐based electrolyte.

An Aurbach‐type partial‐stripping protocol was further employed to minimize the uncertainty associated with the Al surface by exclusively evaluating the effect of electrode‐electrolyte interphase on Na plating/stripping (Figure 3g). A Na reservoir is first created on the current‐collector working electrode, and a smaller fixed capacity is then cycled for n cycles while keeping the working electrode in a metal‐covered state. After cycling, the remaining reservoir is exhaustively stripped to a predefined cut‐off cell voltage, and the residual charge is quantified. The average CE was calculated as follows [35]:

(5) $$\begin{array}{c} \;CE_{\mathrm{avg}}=\frac{nQ_{C}+Q_{S}}{nQ_{C}+Q_{T}}\;,\; \end{array}$$

where *Q_T_* is the initial Na reservoir capacity (5.0 mAh cm^−2^), *Q_C_* is the cycled capacity in each partial plating/stripping step (1.0 mAh cm^−2^), *n* is the number of partial cycles (10), and QS is the residual Na capacity obtained from the final exhaustive stripping step to 1.0 V. Based on this evaluation, the pDFHA@Al electrode delivered a superior average Coulombic efficiency (CE_avg_) of 99.7%, which is significantly higher than that of bare Al (99.6%). This enhanced efficiency indicates that the pDFHA layer effectively suppresses irreversible Na consumption and stabilizes the interphase during repeated deposition/stripping. A quantitative summary of the key electrochemical and interfacial parameters discussed above is provided in Table S1.

Figure 3h presents a comprehensive comparison between this work and representative interface‐modification strategies for Na metal anodes in terms of three key parameters (i.e., current density, per‐cycle areal capacity, and cycling time). The detailed literature values used for this comparison are listed in Table S2. Under matched current‐density and areal‐capacity conditions, the Na||pDFHA@Al symmetric cell sustains stable plating/stripping for over 2000, 1000, and 500 h at 1, 2, and 5 mA cm^−2^ with corresponding per‐cycle areal capacities of 1, 2, and 5 mAh cm^−2^, respectively, outperforming representative literature reports under the corresponding operating conditions. This wide‐current durability is consistent with the mitigated polarization and improved Na^+^ transport across the pDFHA‐derived interphase.

**FIGURE 4:**
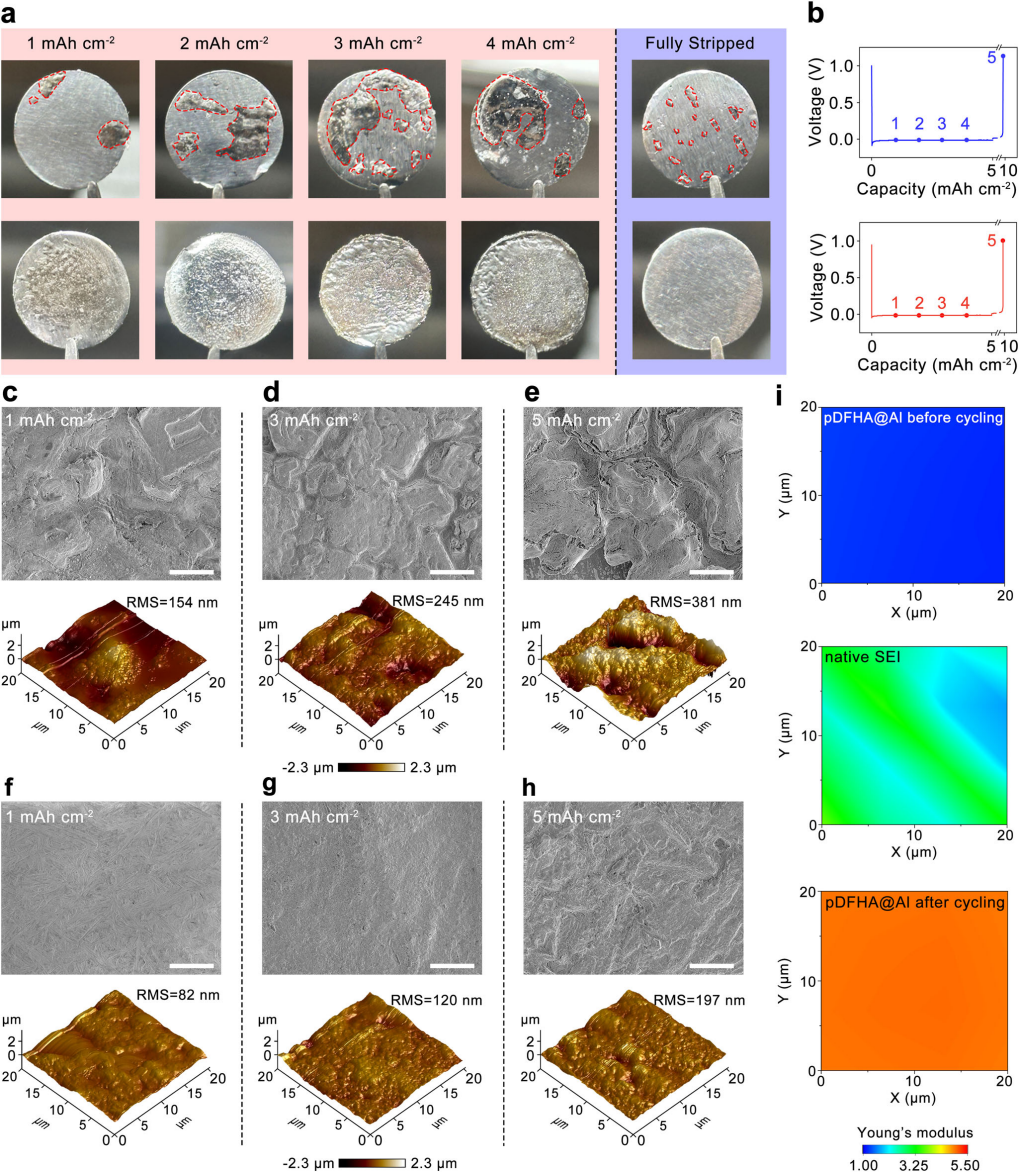
Morphological evolution of Na deposition on bare Al and pDFHA@Al electrodes. (a) Optical photographs of bare Al (top) and pDFHA@Al (bottom) collected during plating (snapshots 1–4 at 1, 2, 3, and 4 mAh cm^−2^, respectively) and after full stripping (snapshot 5), together with (b) the corresponding galvanostatic voltage‐capacity profiles. Red dashed outlines in (a) highlight nonuniform Na deposition on bare Al. (c–e) Morphological characterization of Na deposition on bare Al at (c) 1, (d) 3, and (e) 5 mAh cm^−2^. (f–h) Morphological characterization of Na deposition on pDFHA@Al at (f) 1, (g) 3, and (h) 5 mAh cm^−2^. In each panel, top rows show top‐view SEM images (scale bars: 10 µm) and bottom rows show 3D AFM topography maps (scan area: 20 µm × 20 µm). (i) Two‐dimensional Young's modulus maps of different interphases.

To understand the origin of the superior electrochemical reversibility of the pDFHA‐modified electrode, the Na^+^ plating behavior was systematically investigated from millimeter‐scale to micro‐scale. First, the distribution uniformity of Na plating/stripping at millimeter scale on bare Al (top) and pDFHA@Al (bottom) were monitored as indicated in Figure 4a. Specifically, four representative plating status (1, 2, 3, and 4 mAh cm^−2^) together with the fully stripped status were selected for monitoring as marked by blue and red points in Figure 4b. For bare Al, spatially pronounced nonuniform distribution of plated Na (outlined by red dashed lines) is observed at each of the capacity points (1−4), indicating heterogeneous Na^+^ deposition on the bare Al electrode. After full stripping to the cut‐off voltage (5), multiple discrete residues remain on the bare Al surface, supporting the non‐uniform Na consumption during cycling. In contrast, the pDFHA@Al electrode exhibits a comparatively continuous and uniform surface texture throughout the entire plating process up to 4 mAh cm^−2^, suggesting that the pDFHA interphase facilitates a more homogenized distribution and utilization of plated Na on the current collector. Furthermore, the surface of pDFHA@Al electrode recovers its original clean appearance after full stripping, demonstrating that the pDFHA‐derived interphase enables a more uniform Na consumption at the current collector scale.

The surface morphology evolution of plated Na at micro‐scale on bare Al and pDFHA@Al was examined by acquiring top‐view SEM images after Na^+^ plating to identical areal capacities of 1, 3, and 5 mAh cm^−2^ (left/middle/right). In the top‐view SEM images of bare Al (Figure 4c–e, upper rows), the surface contrast remains spatially nonuniform across the entire capacity range, accompanied by irregular micron‐scale domains and ridge‐like boundary features, while the surface height heterogeneity increases markedly as Na accumulates. As the areal capacity increases from 1 to 3 mAh cm^−2^, these relief features and boundary characteristics become progressively more pronounced. At 5 mAh cm^−2^, the locally elevated regions further intensify, and distinct valleys appear between them. The 3D AFM topographic images of the electrodes at corresponding areal capacities over 20 µm × 20 µm were extracted to quantify the surface morphological evolution. The bare Al AFM images (Figure 4c–e, lower rows) show that localized mound‐like protrusions at 1 mAh cm^−2^ develop into more irregular structures at 3 mAh cm^−2^ and further evolve into a landscape featuring a pronounced depression surrounded by raised ridge‐like features at 5 mAh cm^−2^. Accordingly, the RMS roughness increases from 152 nm to 245 nm and 381 nm as the areal capacity increases from 1 to 3 and 5 mAh cm^−2^, respectively.

By contrast, pDFHA@Al exhibits a comparatively more continuous morphology with finer textures across the same capacity range (Figure 4f–h). Although the surface becomes slightly rougher as capacity increases from 1 to 5 mAh cm^−2^, no pronounced inter‐domain contrast or coarse boundary features are observed. AFM maps reveal more moderate height variations across all three capacities with a highly concentrated height range (mostly within ±0.5 µm) compared to the broad spread spanning the full ± 1.0 µm window of the bare Al. The RMS roughness is effectively restrained to 82, 120, and 197 nm at 1, 3, and 5 mAh cm^−2^, respectively.

To directly support the mechanical robustness and elastic compliance of the pDFHA‐derived interphase, AFM nanomechanical measurements were added, as shown in Figures 4i and S17a. Before cycling, pDFHA@Al exhibits a Young's modulus of approximately 1.2 GPa, which is much lower than that of the native SEI on pristine Al (3.1 GPa), indicating the intrinsically more compliant nature of the pure pDFHA layer. Note that the modulus distribution of pDFHA@Al is substantially more uniform than that of the native SEI, suggesting that the iCVD‐deposited pDFHA coating provides a laterally homogeneous mechanical environment, whereas the native SEI is characterized by pronounced spatial heterogeneity. After cycling, the pDFHA@Al‐derived interphase exhibits the highest Young's modulus of 4.9 GPa, indicating improved elastic recovery and resistance to irreversible deformation. The load–displacement curves of different interphases (Figure S17b–d) further suggest that the cycled pDFHA@Al‐derived interphase undergoes predominantly reversible elastic deformation with minimal hysteresis, whereas both the pristine pDFHA layer and the native SEI exhibit more pronounced loading–unloading separation and residual deformation. These results confirm that the pDFHA+NaF‐derived interphase exhibits higher stiffness, better elastic recovery, and improved lateral mechanical uniformity than the native SEI and pDFHA alone.

Overall, these SEM and AFM analyses indicate that pDFHA@Al supports a more uniform deposition mode with suppressed vertical growth.

To gain deeper insight into how the pDFHA‐derived interphase regulates Na^+^ deposition behavior, the chemical composition and spatial organization of the interphase were systematically characterized using depth‐resolved XPS after initial cycling. In particular, the naturally formed SEI on bare Al and the pDFHA‐derived interphase on pDFHA@Al were comparatively analyzed after initial cycling to elucidate their differences in both chemical constitution and depth‐dependent distribution.

**FIGURE 5:**
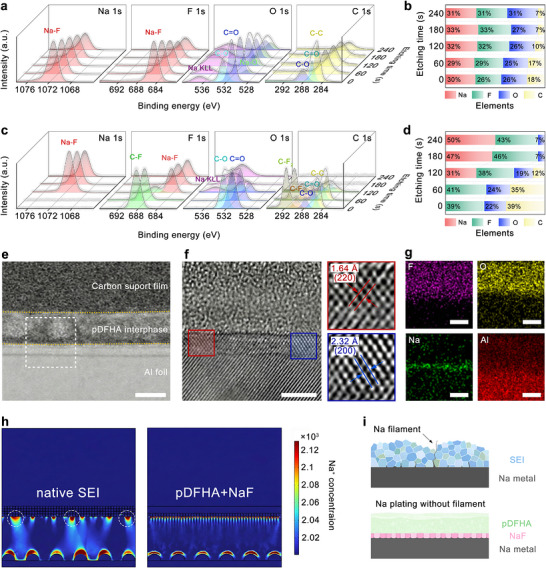
Compositional, structural, and theoretical characterizations of the pDFHA‐derived interphase after initial cycling. (a–d) Depth‐resolved XPS spectra (Na 1s, F 1s, O 1s, C 1s) and corresponding atomic fractions of (a, b) bare Al and (c, d) pDFHA@Al over 240 s of sputter‐etching. (e–g) Cross‐sectional characterizations of pDFHA@Al after full stripping, including (e) a low‐magnification TEM image of the interphase, (f) high‐resolution TEM image showing lattice spacings of 2.32 and 1.64 Å beneath a region lacking lattice fringes, and (g) the corresponding EDX elemental maps of F, O, Na, and Al. (h) FEA‐simulated Na^+^ flux distributions comparing the native SEI and the pDFHA‐derived SEI. (i) Schematic illustration of the interfacial Na flux. Scale bars: 20 nm in (e) and 10 nm in (f, g).

For the bare Al electrode, the Na 1s spectrum exhibits a strong feature centered at 1071.2 eV with a slightly asymmetric tail toward higher binding energy, as shown in Figure 5a. Such a response has been widely reported in NaPF_6_‐based ether electrolytes and can generally be attributed to Na species coordinated with electronegative anionic environments, such as Na─F and Na─O bonding configurations. Notably, this Na‐related signal remains detectable throughout the entire depth investigated, indicating that these Na‐containing inorganic species are distributed across the analyzed thickness. Consistently, the F 1s spectra show a persistent peak assignable to NaF at all sputtering depths, further confirming the presence of fluorinated inorganic Na species throughout the SEI (Figure 5a). In addition, the O 1s and C 1s spectra are consistent with the characteristic decomposition products commonly reported for NaPF_6_‐containing ether electrolytes, suggesting that the interphase formed on bare Al is a conventional electrolyte‐derived SEI [9]. Besides inorganic species, organic components arising from solvent decomposition are also clearly observed. Further, the presence of a Na─O related contribution in the O 1s region is in good agreement with the Na 1s analysis, further supporting the coexistence of oxide‐ or oxyanion‐containing sodium species, which was typically observed within the native SEI [9, 28, 57, 58, 59].

It is worth noting that the overall intensities of the Na 1s, F 1s, O 1s, and C 1s signals associated with the SEI gradually decrease with increasing sputtering depth, which is reasonably attributed to the progressive removal of the interphase during Ar^+^ etching. However, as shown in Figure 5b, the relative atomic fractions of the major species remain broadly similar throughout the analyzed depth. This behavior suggests that the electrolyte‐derived SEI on bare Al does not exhibit a pronounced depth‐dependent stratification; instead, its organic and inorganic components are spatially intermixed in a relatively disordered manner, which is characteristic of a native SEI formed through stochastic electrolyte decomposition.

In sharp contrast, the pDFHA‐derived interphase displays a distinctly different depth‐dependent chemical distribution as indicated in Figure 5c. In the Na 1s spectra, no discernible Na‐related signal is detected at the surface (0 s) or after 60 s of sputter‐etching, indicating that the outer region of the interphase is essentially free of Na‐containing species. In addition, the F 1s spectra at 0 s and 60 s shows only a single peak located at 688.3 eV, which matches well with the characteristic C‐F bonding environment of the pDFHA film. This observation suggests that the outer portion of the interphase is dominated by the pDFHA polymer layer. This interpretation is further corroborated by the O 1s and C 1s spectra, particularly the C 1s region, in which the characteristic components closely resemble those of the as‐deposited pDFHA film (Figure 2f), confirming that the upper part of the interphase largely retains the chemical signature of the original fluorinated polymer.

With increasing sputtering depth, however, the spectral contributions associated with pDFHA in the F 1s, C 1s, and O 1s regions progressively diminish (Figure 5c). At deeper etched regions, a new and symmetric Na 1s peak emerges at 1071.3 eV. Unlike the broader and slightly asymmetric Na 1s response observed for bare Al, this peak is highly symmetric, indicating that it is dominated by a more chemically uniform Na‐containing species. Enlarged Na 1s spectra for bare Al at 0 s and pDFHA@Al at 180 s are provided in Figure S18, further highlighting the broader, more prominent asymmetric Na 1s response of the native interphase, compared to the symmetric Na 1s feature observed for the pDFHA‐derived interphase. Concurrently, a new F 1s peak appears at 684.4 eV upon further sputtering. Of note, the Na─F component in the pDFHA‐modified sample shows a depth‐dependent shift relative to that of the pristine sample, appearing at lower binding energy at sputtering times of 120 s and 240 s. This behavior is attributed to the distinct local interfacial environments encountered at different depths in the pDFHA‐derived hybrid interphase. Specifically, the 120 s and 240 s regions likely correspond to mixed chemical environments, such as organic/inorganic coexistence and inorganic/metallic Na coexistence, respectively, which can alter the local charge‐compensation behavior during XPS measurement and thereby induce depth‐dependent charging or differential charging effects. In contrast, the native SEI on pristine Al is more laterally random and lacks such a vertically stratified hybrid structure, leading to better peak‐position consistency across different sputtering depths. In addition, the Na KLL feature in the O 1s region is consistently shifted to higher binding energy in the pDFHA‐modified sample at 120, 180, and 240 s, indicating that this shift mainly originates from the difference in Na‐related chemical environments between the native SEI and the pDFHA‐derived interphase. In the native SEI, the Na KLL signal is influenced by multiple Na‐containing species, such as Na_2_O, NaOH, Na_2_CO_3_, and sodium alkyl carbonates, whereas in the pDFHA‐derived interphase it is dominated primarily by NaF. Combined with the Na 1s analysis, this feature can be assigned to Na–F bonding, indicating the formation of a NaF‐rich inorganic domain beneath the pDFHA layer and thereby confirming the hybrid nature of the interphases.

The depth‐dependent relative atomic fractions shown in Figure 5d further confirm the spatial compositional distribution within the pDFHA‐derived interphase. The near‐surface region (0–60 s) contains only C, O, and F species, consistent with a pDFHA‐rich polymer outer layer. By contrast, the deeper region (180–240 s) is characterized predominantly by Na and F, indicating the formation of a NaF‐rich inorganic inner layer through interfacial conversion. Together, these findings reveal that the pDFHA‐derived interphase is a vertically integrated bilayer hybrid architecture rather than a randomly mixed interphase, comprising an outer polymer‐rich domain and an inner inorganic‐rich domain, which is fundamentally different from the compositionally disordered native SEI formed on bare Al.

To more clearly resolve the structural features of the pDFHA‐derived interphase, a fully stripped pDFHA@Al electrode was selected for post‐mortem characterization. This electrode configuration eliminates interference from residual metallic Na and therefore enables a more reliable examination of the intrinsic interphase architecture after Na stripping. To directly visualize the vertical stratification inferred from the depth‐resolved XPS analysis, cross‐sectional TEM coupled with EDX mapping was performed on the fully stripped pDFHA@Al electrode (Figure 5e–g). As shown in the low‐magnification TEM image (Figure 5e), a continuous interphase layer with a uniform thickness of approximately 20 nm is conformally coated on the Al substrate beneath the carbon support film. This interphase is attributed to the pDFHA film. To further elucidate the local crystallographic characteristics of this interphase, high‐resolution transmission electron microscopy (HRTEM) was acquired from the region marked by the white square in Figure 5e. As shown in Figure 5f, the upper portion of the interphase does not exhibit discernible lattice fringes, consistent with an amorphous pDFHA outer layer. This assignment is further supported by the x‐ray diffraction (XRD) pattern of a reference pDFHA film (Figure S19), which shows no distinct diffraction peaks, in line with prior reports on related fluoroalkyl acrylate polymers [60, 61]. In contrast, the bottom region near the substrate shows highly ordered lattice fringes corresponding to crystalline Al. More importantly, at the interface between the amorphous outer layer and the Al substrate, an additional crystalline region with lattice orientations distinct from those of the Al substrate can be clearly identified, indicating the formation of an interfacial inorganic nanolayer rather than merely an extension of the underlying Al lattice.

The enlarged regions marked by the red and blue squares reveal lattice spacings of 1.64 and 2.32 Å, which can be indexed to the (220) and (200) planes of cubic NaF, respectively [62, 63]. These results provide direct crystallographic evidence for the presence of NaF, located at the interface between the Al substrate and the overlying amorphous pDFHA‐rich layer, with an estimated thickness of approximately 4 nm. The corresponding EDX elemental maps (Figure 5g) further clarify the chemical stratification across the pDFHA‐derived interphase. The F signal is distributed throughout the entire interphase thickness, suggesting that fluorine is present in both the outer fluorinated polymer layer and the inner inorganic region. The O signal is primarily localized in the outer region of the interphase, consistent with the presence of the oxygen‐containing ester backbone of pDFHA and thus further supporting the assignment of the outer layer as polymer‐rich. It should be noted that because a fully stripped electrode was used for the TEM and EDX analyses, the detected Na signal cannot originate from residual metallic Na deposits; instead, it should be attributed to Na‐containing interphase species. Importantly, the Na signal is concentrated in a narrow inner band directly above the Al substrate, which, together with the HRTEM lattice‐fringe analysis, provides compelling evidence that the inorganic inner layer is predominantly composed of NaF. Taken together, the pDFHA‐derived interphase is not a randomly intermixed SEI, but a vertically stratified hybrid interphase consisting of an amorphous pDFHA‐rich outer layer and a crystalline NaF‐rich inner layer at the Al surface.

To further elucidate the impact of lateral compositional homogeneity on Na^+^ plating behavior, two computational models with identical interphase thickness were constructed as shown in Figure S20. The representative model geometries were designed based on the experimentally observed surface texture of bare Al revealed by the top‐view SEM and AFM analyses (Figures 2c and S1), and the simulation parameters are summarized in Table S3. In the first model, randomly distributed compositional domains were introduced within the interphase to represent the heterogeneous native SEI structure (Figure 5h). Because different phases exhibit distinct Na^+^ transport kinetics, spatial variations in ionic conductivity and migration barriers result in non‐uniform Na^+^ flux distribution during plating. As shown in Figure 5h, regions with comparatively faster ion transport give rise to locally amplified Na^+^ flux beneath the interphase. These localized high‐flux regions act as electrochemical “hotspots,” promoting preferential Na accumulation and the formation of Na filaments. Such filament growth increases the likelihood of partial electronic pathways, ultimately leading to soft short circuit behavior. In contrast, the pDFHA‐derived interphase, characterized by horizontally uniform composition, exhibits more consistent Na^+^ transport properties across the interface. Under identical thickness conditions, the spatial distribution of Na^+^ flux remains comparatively homogeneous, which significantly mitigates local current amplification and suppresses hotspot formation, leading to the uniform growth of the plated Na. As a result, the probability of filament‐induced soft short circuits is markedly reduced. These results also rationalize why lateral compositional uniformity was screened as a primary design criterion: even under identical thickness, lateral chemical heterogeneity alone is sufficient to induce Na^+^ flux localization and filament initiation. As schematically illustrated in Figure 5i (top), for the electrolyte‐derived SEI, lateral heterogeneity in interfacial kinetics gives rise to spatially nonuniform Na^+^ transport and deposition behavior. Regions with more favorable Na^+^ migration kinetics or a locally thinner SEI are more susceptible to preferential Na nucleation and growth, leading to localized accumulation of Na^0^. Once such preferential deposition is initiated, these sites become increasingly prone to continued Na accumulation during subsequent cycling because the amplified local electric field and ion flux further reinforce site‐selective growth, thereby creating a self‐accelerating feedback loop. As a result, Na deposits progressively propagate through defects or weakly protected regions within the SEI and eventually evolve into Na filaments. When these filaments electronically connect the two electrodes, soft short circuiting is triggered. In contrast, the pDFHA‐derived interphase exhibits a laterally homogeneous compositional distribution (Figure 5i, bottom), consisting of an ultrathin organic‐inorganic hybrid architecture with uniformly distributed amorphous pDFHA and NaF nanograins. Such horizontal compositional homogeneity minimizes spatial variations in Na^+^ migration kinetics and thus homogenizes the interfacial Na^+^ flux across the electrode surface. Consequently, Na deposition proceeds with comparable magnitude and morphology at different lateral positions, without generating the pronounced local growth disparity required for filament initiation. This effectively suppresses Na filament formation, thereby preventing the initiation of soft short circuits and consequently avoiding the subsequent degradation associated with soft shorts as well as the eventual evolution into hard short circuits. Therefore, these results verify that the pDFHA‐derived interphase, featuring a laterally compositionally homogeneous, ultrathin organic‐inorganic hybrid structure, enables soft‐short‐free Na deposition and serves as an ideal interphase for stable Na metal anodes.

**FIGURE 6:**
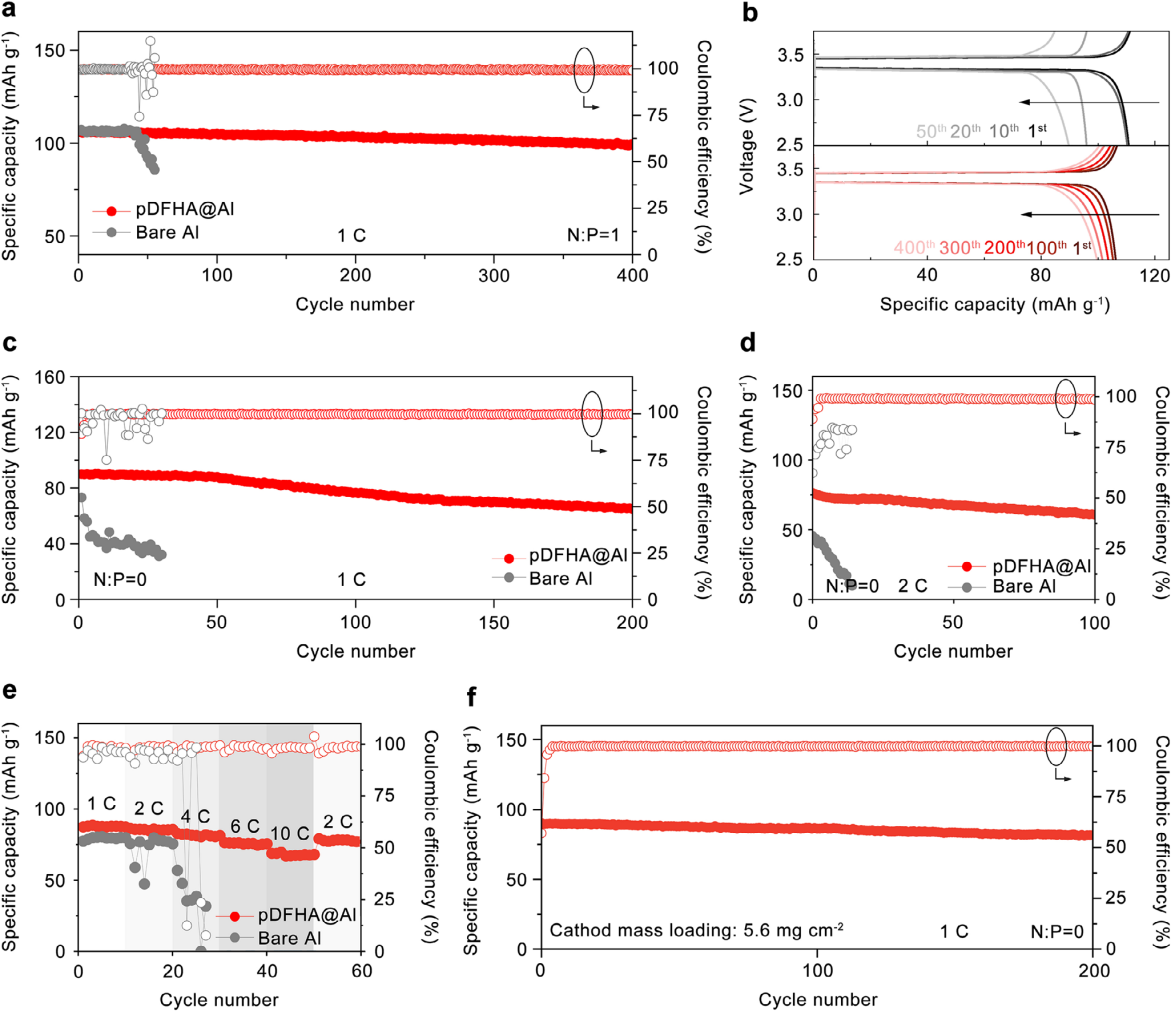
Full cell evaluation. (a) Cycling performance of Na@bare Al||NVP and Na@pDFHA@Al||NVP full cells at 1 C (N:P ratio = 1). (b) Voltage‐capacity profiles of bare Al||NVP and pDFHA@Al||NVP with an N:P ratio of 1 at different cycles under 1 C. (c) Cycling performance of bare Al||NVP and pDFHA@Al||NVP under 1 C with an N:P ratio of 0. (d) Cycling performance of bare Al||NVP and pDFHA@Al||NVP full cells (N:P ratio = 0) at 2 C. (e) Rate performance of bare Al||NVP and pDFHA@Al||NVP (N:P ratio = 1) cells cycling at increasing rates from 1 to 10 C. (f) Cycling performance of the pDFHA@Al||NVP cell (N:P ratio = 0) with a high areal loading of 5.6 mg cm^−2^ at 1 C.

To evaluate the effectiveness of the pDFHA@Al current collector from a practical application perspective, NVP full cells with different N:P ratios were assembled. First, Na@bare Al||NVP and Na@pDFHA@Al||NVP cells with an N:P ratio of 1 were manufactured by pre‐plating Na and evaluated at 1 C (1 C = 117 mAh g^−1^). As shown in Figure 6a, although both cells exhibited a similar initial CE (99.6%), a clear difference was observed in long‐term cycling stability. The Na@bare Al||NVP cell recorded an initial discharge capacity of 110.8 mAh g^−1^ and remained largely unchanged through the 20th cycle, but then deteriorated rapidly, decreasing to 96.0 mAh g^−1^ at the 50th cycle and effectively failing within approximately 55 cycles. In contrast, the Na@pDFHA@Al||NVP cell retained 99.6 mAh g^−1^ after the 400th cycle from an initial discharge capacity of 106.2 mAh g^−1^, corresponding to a capacity retention of 93.8%, while its CE remained stable with an average value of 99.9% over 400 cycles. These results demonstrate that the pDFHA interphase effectively improves Na reversibility and stabilizes full‐cell operation under anode conditions. The charge/discharge voltage profiles at selected cycles (Figure 6b) further support this trend, as the bare Al‐based cell exhibited a markedly shortened charge/discharge plateau by the 50th cycle, whereas the pDFHA@Al‐based cell largely preserved its overall profile shape up to the 400th cycle.

Subsequently, anode‐free NVP full cells using bare Al and pDFHA@Al without Na pre‐plating were evaluated (Figure 6c). In the anode‐free configuration, because no pre‐stored Na reservoir exists on the Al side, CE fluctuation may accompany the formation of reversible Na inventory during the initial few cycles. Under these conditions, the bare Al||NVP cell recorded an initial discharge capacity of 73.4 mAh g^−1^ at 1 C, but suffered severe performance decay within approximately 30 cycles, accompanied by pronounced CE fluctuation and a decrease in discharge capacity to 38.4 mAh g^−1^ at the 20th cycle. In contrast, the pDFHA@Al||NVP cell delivered an initial discharge capacity of 90.1 mAh g^−1^ with an initial CE of 89.3%, after which the CE rapidly stabilized to an average CE of 99.7% during the cycling time, indicating highly reversible Na plating/stripping after the initial cycles, while a reversible capacity of 65.1 mAh g^−1^ was maintained even after the 200^th^ cycle, corresponding to a capacity retention of 72.2%. This suggests that the pDFHA interphase mitigates initial Na loss and induces more stable Na plating/stripping under anode‐free conditions.

The charge/discharge voltage profiles under anode‐free 1 C conditions are shown in Figure S21. The bare Al||NVP cell exhibited a rapid decrease in discharge capacity together with progressively shortened charge/discharge plateaus upon cycling, which is consistent with continuous cyclable Na loss under anode‐free conditions. In contrast, the pDFHA@Al||NVP cell maintained longer plateaus and lower voltage hysteresis up to the 200th cycle, indicating more stable interfacial Na plating/stripping behavior.

The cycling stability of the anode‐free full cells was further evaluated at 2 C (Figure 6d). After showing a discharge capacity of 88.7 mAh g^−1^ and a CE of 86.9% in the activation cycle, the pDFHA@Al||NVP cell recorded a discharge capacity of 90.6 mAh g^−1^ in the first cycle and still retained 74.4 mAh g^−1^ after the 100th cycle, corresponding to a capacity retention of 82.1% based on the first cycle. In addition, the CE increased to 99.7% in the 10th cycle and then stabilized in an average value of 99.6%. In contrast, the bare Al||NVP cell delivered an initial discharge capacity of 70.1 mAh g^−1^ and an initial CE of 72.5% but then degraded rapidly to 27.1 mAh g^−1^ at the 20th cycle and effectively failed within approximately 25 cycles. During this period, the CE of the bare Al||NVP cell remained below 90.0% throughout cycling. The voltage profiles at selected cycles (Figure S22) showed the same tendency, with more rapid plateau shortening in the bare Al‐based cell, whereas the pDFHA@Al‐based cell maintained a more stable profile evolution up to the 100th cycle.

To further assess long‐term stability at higher current density, the anode‐free pDFHA@Al||NVP cell was also evaluated at 5 C (Figure S23). The pDFHA@Al||NVP cell delivered an initial discharge capacity of 76.6 mAh g^−1^ with an initial CE of 89.0% and still retained 60.8 mAh g^−1^ after the 100th cycle, corresponding to a capacity retention of 80.3%. The CE also reached 99.4% and 99.6% at the 25th and 50th cycles, respectively. The corresponding voltage profiles (Figure S24) further show that the pDFHA@Al‐based cell maintained relatively distinct charge/discharge plateaus even under high‐rate conditions.

In addition, the rate capability of bare Al||NVP and pDFHA@Al||NVP cells was compared under stepwise current rates of 1, 2, 4, 6, and 10 C, followed by returning to 2 C (Figure 6e). The pDFHA@Al||NVP cell delivered 10‐cycle mean specific capacities of 87.7, 85.5, 81.5, 75.7, and 67.9 mAh g^−1^, respectively, and recovered a capacity of 77.9 mAh g^−1^ when the rate was returned to 2 C. In contrast, the bare Al||NVP cell exhibited mean capacities of 79.5 and 72.1 mAh g^−1^ with lower average CEs of 95.6% and 93.3% at 1 C and 2 C, respectively, and both capacity and efficiency deteriorated sharply at higher rates above 4 C. These results indicate that the pDFHA interphase effectively improves not only long‐term cycling stability but also Na plating/stripping kinetics under high‐rate conditions.

Finally, to evaluate the practical applicability under a more stringent condition, full cells were tested with a high NVP cathode loading of 5.6 mg cm^−2^ (Figure 6f). The cell maintained stable cycling for 200 cycles, delivering an average CE of 99.7% and a capacity retention of 90.8%. These results further demonstrate the excellent stability of the pDFHA interphase and support the promise of pDFHA@Al as a current‐collector strategy for anode‐free NVP full cells. Moreover, as summarized in Table S4, the NVP full‐cell performance achieved in this work demonstrates the practical promise of the pDFHA‐derived interphase for applications requiring high energy density and is highly competitive with previously reported sodium metal and anode‐free sodium full cells based on interphase‐engineering strategies, particularly in terms of cycling stability, rate capability, and high‐loading operation. To further assess the practical applicability of the pDFHA‐modified electrode under large‐format conditions, pouch full cells based on a pre‐sodiated pDFHA‐modified anode were additionally assembled and evaluated. As shown in Figure S25, the pouch cell delivered an initial energy density of 180.4 Wh kg^−1^ at 0.1 C. As summarized in Table S5, this value is higher than or comparable to that of most previously reported pouch‐type sodium‐based cells, further demonstrating the practical promise of the pDFHA‐derived interphase.

## 3 Conclusion

This work establishes interphase design principles for realizing soft‐short‐circuit‐free anodes in anode‐free sodium metal batteries by identifying three essential criteria: (1) lateral compositional homogeneity to eliminate spatial variations in Na^+^ migration kinetics and thereby suppress localized Na^+^ flux amplification, (2) ultrathin thickness (< 70 nm, considering native SEI) to minimize Na^+^ transport distance across the interphase and reduce kinetic polarization, and (3) an organic‐inorganic hybrid architecture to accommodate interfacial deformation while maintaining sufficient mechanical robustness against filamentary Na growth. Distinct from previous interface modification studies that mainly demonstrate the effectiveness of specific materials or strategies, this work further shows that soft‐short‐free operation in anode‐free Na batteries should be actively controlled through a mechanism‐guided interphase design framework. Guided by these criteria, a uniform 20 nm pDFHA layer was deposited on Al current collectors via iCVD. Upon the initial Na plating process, an approximately 4 nm NaF‐rich inorganic layer is formed in situ at the interface between the iCVD interphase and Al, while the remaining pDFHA serves as a compliant polymer‐rich outer framework, together giving rise to a laterally homogeneous organic‐inorganic hybrid interphase, as verified by XPS depth profiling and cross‐sectional TEM analyses.

Multiscale characterization from millimeter‐scale optical observations to micrometer‐scale SEM analyses reveals that the pDFHA‐derived interphase not only promotes a more spatially uniform distribution and consumption of plated Na across the current collector but also produces a smoother and more homogeneous plating morphology. Consequently, the modified Al electrode exhibits exceptional electrochemical stability, sustaining over 2000 h of stripping/plating in symmetric cells at 1 mA cm^−2^ with a cutoff capacity of 1 mAh cm^−2^ without observable soft short behavior. In contrast, the bare Al electrode exhibits soft short signatures beginning at about 130 h and ultimately undergoes hard short failure at approximately 285 h.

FEA further elucidated the mechanism underlying soft short suppression by the established hybrid interphase. Under identical thickness conditions, randomly distributed compositional domains, which represent native SEI structures, induce localized Na^+^ flux concentration and current amplification. In contrast, the horizontally uniform hybrid interphase redistributes Na^+^ flux more evenly, mitigating localized hotspots that initiate soft short circuits. To evaluate practical applicability, anode‐free full cells paired with NVP cathodes deliver 90.6 mAh g^−1^ with 82.1% capacity retention even under 2 C, significantly outperforming pristine counterparts. At higher mass loading (5.6 mg cm^−2^), corresponding to practical areal capacities, the modified system maintains 90.1 mAh g^−1^ with 90.8% retention after 200 cycles at 1 C, demonstrating robust performance under realistic operating conditions.

Overall, this work establishes a practical interphase design principle for mitigating soft short circuits in anode‐free sodium metal batteries under application‐relevant conditions, and the demonstrated design framework may be broadly extended to other metal‐anode systems toward soft‐short‐free operation.

## Author Contributions

**Yeongjun Oh**: conceptualization, methodology, investigation, formal analysis, data curation, writing – original draft, writing – review and editing, visualization, validation. **Yuxuan Zhang**: conceptualization, methodology, investigation, writing – original draft, writing – review and editing, visualization. **Jinwook Baek**: validation, writing – review and editing. **Minyoung Kim**: validation, writing – review and editing. **Jeongmin Park**: investigation, resources, formal analysis. **Myung Gyu Lee**: formal analysis, resources, investigation. **Chung Soo Kim**: resources, supervision. **Sunghwan Lee**: conceptualization, supervision, project administration, funding acquisition, writing – review and editing.

## Conflicts of Interest

The authors declare no conflicts of interest.

## Supporting information




**Supporting File 1**: anie73060‐sup‐0001‐SuppMat.docx.

## Data Availability

The data that support the findings of this study are available from the corresponding author upon reasonable request.
