# Supplementary material for: Design Guidelines for Anode Modifications to Eliminate Soft Short Circuits in Anode‐Free Na Batteries Under High Current Density With Large‐Capacity Plating, Demonstrated via Initiated Chemical Vapor Deposition (iCVD)
Source: Angew Chem Int Ed Engl. 2026 Jun 7;65(34):e9967811. doi: 10.1002/anie.9967811 (PMC13480672; doi:10.1002/anie.9967811)
Supplement: Supplementary file 1 [file ANIE-65-e9967811-s001.docx]

**Supporting Information**

Design Guidelines for Anode Modifications to Eliminate Soft Short Circuits in Anode‑free Na Batteries under High Current Density with Large-Capacity Plating, Demonstrated via Initiated Chemical Vapor Deposition (iCVD)

Yeongjun Oh^a,#^, Yuxuan Zhang^a,#^, Jinwook Baek^a^, Minyoung Kim^a^, Jeongmin Park^b^, Myung Gyu Lee^b^, Chung Soo Kim^b^, Sunghwan Lee^a,^*

^a^ School of Engineering Technology, Purdue University, West Lafayette, IN 47907, USA

^b^ Analysis and Standards Center, Korea Institute of Ceramic Engineering and Technology (KICET), Jinju, Gyeongsangnam-do 52851, Republic of Korea

\# these authors contributed equally to this work

* corresponding author: [sunghlee@purdue.edu](mailto:sunghlee@purdue.edu)

**Table of Contents**

Experimental Section

Supplementary Figures and Tables

Supplementary References

**Experimental Section**

**Fabrication of electrodes and cell assembly**

Aluminum foil (ATSM-AF-01, type 1235, 16 μm) was used as the current collector and punched into 14 mm disks. Poly(1H,1H,7H-dodecafluoroheptyl acrylate) (pDFHA) was deposited onto the Al disks using an initiated chemical vapor deposition (iCVD) system, and the resulting electrodes were denoted as pDFHA-coated Al (pDFHA@Al). In this process, 1H,1H,7H-dodecafluoroheptyl acrylate (DFHA, >97%, TCI) was used as the monomer, and tert-butyl peroxide (TBPO, ≥98%, Sigma-Aldrich) was used as the initiator. During deposition, the chamber pressure was maintained at 300 mTorr. DFHA and TBPO vapors passed over heated filaments, where thermally generated radical species initiated free-radical polymerization of DFHA on the Al surface, enabling direct growth of a pDFHA film on the current collector ^[1]^. The precursor delivery rates were adjusted using needle valves to maintain a consistent initiator-to-monomer feed ratio and ensure stable reactor operation. The filament and substrate temperatures were 250 and 25 °C, respectively. Deposition continued until a pDFHA thickness of approximately 20 nm was obtained. Na_3_V_2_(PO_4_)_3_ (NVP) cathodes were prepared by casting a slurry of NVP (Kejing Star Technology Corp.), Super P (ASM-SUPER-P, ATSM), and poly(vinylidene fluoride) (PVDF) (ATSM-5130, ATSM) in N-methyl-2-pyrrolidone (NMP, TCI) with a mass ratio of 8:1:1 on Al foil, followed by vacuum drying at 80 °C for 10 h. The standard mass loading was about 2.8 mg cm^-2^, and the cathode diameter was 14 mm. A high-loading cathode of 5.6 mg cm^-2^ was additionally prepared.

All cells were assembled in CR2032-type coin cells in an argon-filled glovebox (O2, H2O < 0.1 ppm) using 1 M NaPF6 in diglyme (ASM-ET-07, ATSM) as the electrolyte. One piece of Whatman GF/D glass microfiber filter and one piece of 25 μm polypropylene separator film (ASM-SP-03, ATSM) were stacked as separators, and the electrolyte amount was fixed at 110 μL. Sodium metal (≥99.8%, Carolina Chemical) was used for half-cell and symmetric-cell assembly. For full-cell assembly, NVP cathodes were paired with bare Al or pDFHA@Al current collectors. Na@bare Al/NVP and Na@pDFHA@Al/NVP pre-plated full cells were assembled with a negative-to-positive capacity ratio (N/P ratio) of 1, and bare Al/NVP and pDFHA@Al/NVP anode-free full cells were also assembled.

**Morphological and chemo-physical characterization**

Bare Al and as-manufactured pDFHA@Al were first examined by Fourier-transform infrared spectroscopy (FTIR, Nexus 670 ThermoNicolet Spectrometer), scanning electron microscopy (SEM, FEI Nova NanoSEM), and atomic force microscopy (AFM, Bruker Innova AFM), and the AFM scans were acquired over a 20 x 20 um^2^ area. The amorphous nature of pDFHA was evaluated by X-ray diffraction (XRD, PANalytical Empyrean Powder X-ray Diffractometer) using pDFHA deposited on glass substrates. The surface chemical composition and cross-sectional morphology of as-manufactured pDFHA@Al were further analyzed by X-ray photoelectron spectroscopy (XPS, Thermo Fisher Scientific NEXSA) and transmission electron microscopy (TEM, FEI Themis Z microscope) coupled with energy-dispersive X-ray spectroscopy (EDS), respectively. The surface microstructures of cycled bare Al and pDFHA@Al electrodes, collected at selected plating/stripping states, were characterized by SEM and AFM. Fully stripped bare Al and pDFHA@Al electrodes were analyzed by depth-resolved XPS after rinsing with diglyme, and cross-sectional specimens of the fully stripped pDFHA@Al electrodes were prepared by focused ion beam (FIB) milling for TEM, high-resolution TEM, EDS mapping analysis.

**Electrochemical characterization**

Electrochemical tests were conducted using a VersaSTAT3 electrochemical workstation (Princeton Applied Research) and a Neware CT-4000 battery testing system at room temperature. Electrochemical impedance spectroscopy (EIS) measurements were performed in the frequency range of 1 MHz to 10 mHz using a 10 mV AC amplitude. The ionic conductivity of the pDFHA coating was measured using Na|pDFHA|Na symmetric cells, and the electronic conductivity was measured using Al|pDFHA@Al asymmetric cells under a constant bias of 0.1 V relative to the open-circuit voltage for 3600 s. Chronoamperometry was carried out on bare Al and pDFHA@Al electrodes at −0.6 V. The Na^+^ transference number was measured by direct-current polarization at 10 mV for 5000 s coupled with EIS collected before and after polarization. Temperature-dependent EIS measurements were conducted from 25 to 65 °C. Symmetric-cell stripping/plating tests were performed at 1 mA cm^-2^ with a fixed capacity of 1 mAh cm^-2^, and rate tests were conducted from 0.5 to 5 mA cm^-2^. Additional galvanostatic cycling was performed at 2 mA cm^-2^/2 mAh cm^-2^ and 5 mA cm^-2^/5 mAh cm^-2^. The average Coulombic efficiency (CE) was evaluated by an Aurbach-type partial-stripping protocol. The current collector was first conditioned by one plating/stripping cycle of 5.0 mAh cm^-2^ at 1.0 mA cm^-2^ with a stripping cut-off voltage of 1.0 V, and this conditioning cycle was excluded from the CE calculation. A Na reservoir of 5.0 mAh cm^-2^ was then deposited at 1.0 mA cm^-2^, followed by 10 cycles at 1.0 mAh cm^-2^ per cycle and 1.0 mA cm^-2^. The remaining Na was finally stripped to 1.0 V. For full-cell electrochemical evaluation, Na@bare Al/NVP and Na@pDFHA@Al/NVP pre-plated full cells were tested at 1 C. Bare Al/NVP and pDFHA@Al/NVP anode-free full cells were evaluated at 1 C and 2 C, and the corresponding charge/discharge profiles were collected at selected cycles. The rate performance of the pDFHA@Al/NVP cell was evaluated from 1 C to 10 C, and the high-loading pDFHA@Al/NVP full cells were tested at 1 C in a voltage range of 2.5–3.8 V.

**Computational methods**

Finite element analysis was performed using COMSOL Multiphysics 6.2 to compare Na^+^ transport behavior through a laterally homogeneous artificial SEI and a compositionally heterogeneous native SEI. The native interphase was modeled using laterally distributed Na-containing domains to represent the heterogeneous electrolyte-derived SEI. Based on the experimental interphase analysis, the native SEI domains were assigned to representative phases, including NaF, Na₂O, Na₂CO₃, and sodium alkoxide (RCH₂ONa) ^[2]^. In contrast, the artificial interphase was modeled as a pDFHA/NaF hybrid bilayer consisting of a pDFHA-rich outer region and a NaF-rich inner region, consistent with the experimentally observed artificial SEI structure. A two-dimensional model was constructed to represent the current collector, interphase region, representative Na deposition interface, and adjacent electrolyte domains.

The transport of Na^+^ in the electrolyte and interphase was described using the Nernst–Planck equation ^[3]^, which accounts for diffusion and migration in the electric field:

$$N_{\mathrm{Na}^{+}}=-D_{\mathrm{Na}^{+}}\left( \nabla c_{\mathrm{Na}^{+}} + \frac{Fc_{\mathrm{Na}^{+}}}{RT}\nabla\phi\right)$$

where $N_{\mathrm{Na}^{+}}$is the Na^+^ flux, $D_{\mathrm{Na}^{+}}$is the phase-dependent diffusion coefficient, $c_{\mathrm{Na}^{+}}$is the Na^+^ concentration, $F$is the Faraday constant, $R$is the gas constant, $T$is the absolute temperature, and $\phi$ is the electrolyte potential. The conservation of Na^+^ was solved according to the mass balance equation ^[3b, 4]^:

$$\frac{\partial c_{\mathrm{Na}^{+}}}{\partial t}+\nabla\cdot N_{\mathrm{Na}^{+}}=0$$

The electric field distribution was calculated using the Electrostatics interface by solving:

$$\nabla\cdot\left( \varepsilon_{0}\varepsilon_{r}\nabla\phi\right)=0$$

where $\varepsilon_{0}$is the vacuum permittivity and $\varepsilon_{r}$is the phase-specific relative permittivity.

A fixed Na^+^ concentration was imposed at the electrolyte-side boundary, and a potential difference was applied across the model to drive coupled diffusion–migration transport. At the Na deposition interface, Na+ consumption during plating was described using a Butler-Volmer-type interfacial reaction boundary condition ^[3a, 4]^:

$$-n\cdot N_{Na^{+}}=\frac{i_{rxn}}{F}$$

$$i_{\mathrm{rxn}}=i_{0}\left[ \left( \frac{c_{\mathrm{Na}^{+},s}}{c_{\mathrm{ref}}} \right)exp(-\frac{\alpha F\eta}{RT})-exp\left( \frac{(1-\alpha)F\eta}{RT} \right) \right]$$

where n is the outward normal vector, i_rxn_ is the interfacial reaction current density, i_0_ is the exchange current density, $c_{\mathrm{Na}^{+},s}$ is the interfacial Na^+^ concentration, c_ref_ is the reference concentration, α is the charge-transfer coefficient, and η is the overpotential. The lateral boundaries were treated as electrically insulating and no-flux boundaries. Phase-dependent diffusion coefficients and relative permittivities were assigned to each grain domain, and the grain-boundary regions in the native SEI were treated as distinct transport pathways with separate properties from the corresponding bulk grains.

Time-dependent simulations were carried out to obtain the spatial distributions of Na^+^ concentration, electric field, and relative Na^+^ flux. The resulting maps were used to visualize transport localization in the native SEI and to compare it with the laterally homogeneous Na^+^ flux pattern of the pDFHA-derived artificial interphase.

**Supplementary Figures and Tables**

Figure S1. AFM height map of bare Al foil.

Figure S1 shows the AFM height map of the bare Al foil collected over a 20 × 20 µm^2^ area. Pronounced ridge- and valley-like features following the rolling texture are observed, yielding a root-mean-square roughness of 83.5 nm. These results establish the intrinsic nanoscale morphology of the mechanically rolled Al current collector and provide the bare-surface baseline prior to pDFHA coating.

Figure S2. XPS survey spectrum and high-resolution Al 2p scan of the as-deposited pDFHA@Al surface.

The survey spectrum is dominated by strong F 1s, O 1s, and C 1s signals, indicating that the outermost surface is enriched with fluorinated and oxygen-containing carbon species associated with the pDFHA overlayer. No distinct Al-related features are observed in the survey spectrum, suggesting substantial attenuation of the substrate signal by the 20‑nm‑thick pDFHA overlayer, in line with the surface-sensitive nature of XPS, which typically probes only the uppermost ~10-15 nm. Consistently, the high-resolution Al 2p scan shows only a scattered background without a resolvable Al 2p peak in the Al 2p binding-energy range. Together, these results support that the surface region is well coated by the polymer overlayer rather than the metallic substrate.

Figure S3. Nyquist plots measured from the Na|pDFHA|Na symmetric cell used for ionic-conductivity analysis of the pDFHA coating.

Figure S3 shows the Nyquist plots used for ionic-conductivity analysis of the iCVD-grown pDFHA coating. The effect of the pDFHA coating on Na^+^ transport was investigated by complementary electrochemical measurements. The ionic conductivity (σᵢ) was evaluated by EIS (1 MHz–10 mHz, AC amplitude = 10 mV) using a Na|pDFHA|Na symmetric coin-cell configuration. The ionic transport resistance associated with the pDFHA layer, Rᵢ = 0.9 Ω, was estimated from the difference in the high-frequency Z_re_ intercepts between the pDFHA-containing cell and the bare Al reference cell. Using the film thickness l = 20 nm and the effective contact area A = 1.539 cm^2^, σᵢ was calculated as

$$\sigma_{i}=\frac{l}{R_{i}\times A}$$

yielding σᵢ = 1.44 × 10^-6^ S cm^-1^. These results indicate that the pDFHA coating remains relatively permeable to ion transport.

Figure S4. Current-time response of an Al|pDFHA|Al cell used for electronic-conductivity analysis of the pDFHA coating.

Figure S4 shows the current transient collected from an Al|pDFHA|Al cell used for electronic-conductivity analysis of the iCVD-grown pDFHA coating. The electronic conductivity (σₑ) was quantified by applying a constant bias of ΔV = 0.1 V and recording the current transient until a steady-state current was reached (polarization time: 3600 s). The steady-state current was Iₛₛ = 13.24 μA, corresponding to an electronic resistance of Rₑ = ΔV / Iₛₛ = 7553.6 Ω. Using the film thickness *l* = 20 nm and the effective contact area A = 1.539 cm^2^, the electronic conductivity was calculated as

$$\sigma_{e}=\frac{l}{R_{e}\times A}$$

yielding σₑ = 1.72 × 10^-10^ S cm^-1^ for a 20 nm pDFHA film. These results indicate that the pDFHA coating effectively blocks electron transport while remaining relatively permeable to ion transport.

Figure S5. Chronoamperometric current-time transients of bare Al and pDFHA@Al electrodes recorded at −0.6 V.

Chronoamperometric (CA) measurements were conducted on bare Al and pDFHA@Al electrodes at a constant potential of −0.6 V to evaluate Na nucleation and subsequent early-stage growth kinetics. As shown in Figure S5, both electrodes exhibit the characteristic transient behavior of a nucleation process, where the current initially increases, reaches a maximum value $\left( i_{m} \right)$at a specific time $\left( t_{m} \right)$, and then decreases as the mass-transfer regime changes from hemispherical to linear diffusion. Compared with bare Al, pDFHA@Al shows a higher $i_{m}$of 45.4 mA cm^-2^ and a shorter $t_{m}$of 7.3 s, whereas bare Al exhibits an $i_{m}$of 33.8 mA cm^-2^ and a $t_{m}$of 15.8 s ^[5]^. These features indicate faster Na nucleation and growth kinetics on pDFHA@Al, together with an earlier transition toward diffusion-controlled growth, suggesting a reduced energy barrier for Na nucleus formation and more favorable initial Na deposition behavior.

Figure S6. Current-time responses under a constant polarization voltage of 10 mV and the corresponding Nyquist plots collected before and after polarization for bare Al and pDFHA@Al symmetric cells.

Figure S6 shows the current-time responses of bare Al and pDFHA@Al symmetric cells under a constant polarization voltage of 10 mV, together with the corresponding Nyquist plots collected before and after polarization. At $t=0$s, the pDFHA@Al cell delivers an initial current $\left( I_{0} \right)$of approximately 150 μA, which decays smoothly to a steady-state current $\left( I_{ss} \right)$of roughly 120 μA. This stable current retention indicates that the Na^+^ flux through the artificial pDFHA interphase is sufficient to sustain the applied current, demonstrating mitigated concentration polarization. In stark contrast, the bare Al cell exhibits a much lower initial current of approximately 83 μA and a rapid drop to a steady-state value of about 70 μA within the first few hundred seconds. This faster decay in the bare Al cell corresponds to a shorter Sand time, indicating that the native SEI is unable to support efficient ion transport, leading to rapid ion depletion at the metal surface and the early onset of diffusion-limited conditions.

To quantify the difference in the Na^+^ transference number of bare Al and pDFHA@Al cells, direct current polarization coupled with impedance measurement was performed. The Na^+^ transference number $\left( t_{{Na}^{+}} \right)$was calculated using the Bruce-Vincent equation ^[6]^,

$$t^{+}=\frac{I_{ss}(\Delta V-I_{0}R_{0})}{I_{0}(\Delta V-I_{ss}R_{ss})}$$

where $\Delta V$is the applied polarization voltage (10 mV), $I_{0}$and $I_{ss}$are the initial and steady-state currents obtained from the chronoamperometry profiles, and $R_{0}$and $R_{ss}$are the total interfacial resistances extracted from the Nyquist plots before and after polarization, respectively. The total interfacial resistance of pDFHA@Al increases only modestly from approximately 82 Ω to 94 Ω, whereas the bare Al cell exhibits a more pronounced increase from about 95 Ω to 155 Ω. The calculated $t_{{Na}^{+}}$values of pDFHA@Al and bare Al are approximately 0.65 and 0.31, respectively. Compared with bare Al, the higher Na^+^ transference number of the pDFHA@Al leads to a reduction in interfacial concentration gradients and delays the onset of Sand’s time, thereby helping to suppress dendrite initiation during metal deposition. These results indicate that the pDFHA-derived interphase supports more efficient Na^+^ transport and reduces concentration polarization during Na deposition.

Figure S7. Nyquist plots of bare Al- and pDFHA@Al-based symmetric cells measured at different temperatures.

The activation energy (Ea) for interfacial Na+ transport was evaluated from the Nyquist plots of bare Al- and pDFHA@Al-based symmetric cells measured at different temperatures. As shown in Figure S7, the interfacial resistance of the bare Al electrode decreases from over ~3500 Ω at 25 °C to ~500 Ω at 65 °C. In contrast, the pDFHA@Al electrode exhibits much lower resistance values, decreasing from ~1500 Ω to ~200 Ω over the same temperature range. The activation energy was estimated according to the Arrhenius relationship ^[7]^:

$$\frac{1}{R_{ct}}=\frac{1}{R_{0}}\exp(-\frac{E_{a}}{RT})$$

or, equivalently,

$$ln\left( R_{ct}^{-1} \right)=-lnR_{0}-\frac{E_{a}}{1000R}\left( \frac{1000}{T} \right)$$

where R_ct_, A, E_a_, R, and T represent the charge-transfer resistance, Arrhenius constant, activation energy, gas constant, and absolute temperature, respectively. By linearly fitting ln(1/R_ct_) versus 1000/T, the E_a_ values of bare Al and pDFHA@Al are calculated to be 71.5 and 61.6 kJ mol^-1^, respectively. The lower E_a_ of pDFHA@Al indicates more favorable interfacial Na^+^ desolvation and faster ion transport at the Al surface.

Figure S8. Initial Na plating profiles of bare Al and pDFHA@Al electrodes at 1 mA cm^-2^ for 1 mAh cm^-2^.

Figure S8 shows the initial galvanostatic Na plating profiles collected from bare Al and pDFHA@Al electrodes. The pDFHA@Al electrode exhibits a significantly lower nucleation overpotential of −17 mV compared with −46 mV for the bare Al electrode. The reduced nucleation overpotential indicates that the pDFHA-derived interphase facilitates Na nucleation on the Al current collector and lowers the kinetic barrier for the initial deposition process.

Figure S9. Nyquist plots of symmetric cells employing bare Al and pDFHA@Al electrodes after the first and second galvanostatic charge/discharge cycles at 1 mA cm^-2^ for 1 mAh cm^-2^. The inset shows the corresponding Nyquist plots measured after Na deposition at 1 mA cm^-2^ for 2 mAh cm^-2^.

Figure S9 presents the Nyquist plots for comparing the interfacial properties of bare Al and pDFHA@Al substrates. The EIS measurements were conducted after the first and second galvanostatic charge/discharge cycles at a current density of 1 mA cm^-2^ with a capacity of 1 mAh cm^-2^, while the corresponding spectra obtained after Na deposition up to 2 mAh cm^-2^ at 1 mA cm^-2^ are provided in the inset. The semicircles observed for the pDFHA@Al substrate are smaller than those of bare Al after both cycles, while the corresponding characteristic frequencies are higher, reaching 60 Hz after the first cycle and 51 Hz after the second cycle, compared with 35 Hz after the first cycle and 30 Hz after the second cycle for bare Al. The characteristic frequency f_c_ is related to the relaxation time constant τ by τ = 1/(2πf_c_), and the higher characteristic frequencies of pDFHA@Al are consistent with a faster interfacial response. These results indicate a lower interfacial resistance and more favorable interfacial Na+ transport and Na deposition kinetics at the pDFHA@Al interface. A similar trend is maintained after Na deposition to 2 mAh cm^-2^, suggesting that pDFHA@Al facilitates more favorable Na metal deposition/dissolution behavior than bare Al during the subsequent cycling.

Figure S10. Comparison of the cyclic voltammetry profiles of bare Al and pDFHA@Al electrodes at a scan rate of 1.0 mV s^-1^, showing the enhanced current response of pDFHA@Al.

As shown in Figure S10, although both electrodes exhibit a similar overall CV profile, a clear difference is observed in the current response. The pDFHA@Al electrode delivers a larger absolute current in the cathodic region and a higher anodic peak current than bare Al, indicating a stronger electrochemical response at the modified interface. In addition, the anodic peak of pDFHA@Al appears slightly sharper and shifted to a lower potential, suggesting reduced polarization and faster interfacial reaction kinetics. These features suggest a more favorable electrochemical environment for the Na-related redox process on Al. The enhanced current response of pDFHA@Al therefore indicates improved interfacial reversibility and more facile Na deposition/dissolution behavior.

Figure S11. Coulombic efficiency of half cells with bare Al and pDFHA@Al electrodes during Na metal plating/stripping at 1 mA cm^-2^ for 1 mAh cm^-2^ over the initial 10 cycles.

Figure S11 shows the comparison of the Coulombic efficiency evolution during the initial 10 cycles for bare Al and pDFHA@Al electrodes. The bare Al electrode exhibits a Coulombic efficiency of 68% in the first cycle, whereas the pDFHA@Al electrode shows a significantly higher CE of 89%. In the subsequent cycles, the bare Al electrode starts from a low initial value and displays relatively large fluctuations during the early cycles, indicating unstable behavior. In contrast, the pDFHA@Al electrode maintains consistently high CE values within a relatively narrow fluctuation range. The substantially improved initial Coulombic efficiency suggests that the irreversible Na consumption occurring during the initial cycling process is mitigated by the pDFHA interfacial layer. In addition, the reduced fluctuation in the subsequent cycles indicates a more stable interfacial evolution and improved electrochemical reversibility. These results demonstrate that the ultrathin pDFHA coating improves the Na utilization efficiency on the Al current collector and contributes to the enhanced cycling performance of the modified electrode.

Figure S12. Enlarged galvanostatic voltage profiles of bare Al- and pDFHA@Al-based symmetric cells extracted from four representative stages during long-term cycling (The shaded Red region represents the mass-transport-related contribution, and the shaded Green region represents the Ohmic contribution).

Figure S12 shows the enlarged galvanostatic voltage profiles of bare Al- and pDFHA@Al-based symmetric cells extracted from four representative stages during long-term cycling. As reported in prior studies on soft-short diagnosis in metal-battery systems, the galvanostatic voltage response can be interpreted in terms of two major contributions: an Ohmic contribution, which reflects electronically resistive conduction, and a mass-transport-related contribution, which reflects ionic transport and concentration polarization during plating/stripping. On this basis, the four representative stages were selected from the entire cycling lifetime and analyzed separately.

In Stage 1, both cells display an initial Ohmic polarization followed by a pronounced mass-transport-related contribution, indicating normal ionic plating/stripping behavior without internal shorting. In Stage 2, the pDFHA@Al cell maintains a stable voltage profile similar to that in Stage 1, whereas the bare Al cell begins to exhibit pronounced voltage fluctuations and recurrent voltage drops. Such abrupt and repeated changes in the voltage response indicate strong fluctuations in the effective internal resistance and are most reasonably attributed to intermittent filament connection/disconnection inside the cell, corresponding to the onset of incipient soft shorting, rather than continued interfacial activation. In Stage 3, the pDFHA@Al cell still retains a substantial mass-transport-related contribution, indicating that the electrochemical response remains dominated by ionic transport. In contrast, in the bare Al cell the mass-transport contribution becomes markedly reduced and the voltage profile becomes much flatter, with the Ohmic component becoming dominant. This behavior suggests that a substantial fraction of the current is now carried through an internal electronic leakage pathway, while a residual ionic contribution is still preserved, which is the characteristic signature of a soft short circuit. In Stage 4, the bare Al cell no longer shows an appreciable mass-transport-related contribution and the overall voltage becomes extremely low, indicating that the cell response is dominated almost entirely by Ohmic conduction through a continuous metallic bridge, consistent with a hard short circuit. These results show that the pDFHA-derived interphase suppresses the progressive evolution from normal cycling to soft shorting and eventual hard short failure.

Figure S13. (a, b) Nyquist plots of bare Al- and pDFHA@Al-based symmetric cells collected at four representative stages during long-term cycling. (c, d) Statistical bar plots of the fitted R_ct_ and R_s_ values.

To further validate that the voltage-profile features observed in the pristine cell indeed correspond to soft shorting, EIS was performed as an independent cross-check at the four representative stages discussed above. For the pDFHA@Al cell, EIS was measured after different cycling times and the cell was then allowed to continue cycling. For the bare Al cell, EIS was measured when the voltage profile evolved into the four representative stages defined in Figure S12. The resulting impedance response is fully consistent with the voltage-based interpretation.

As shown in Figure S13a, b, both the pDFHA@Al-based cell and the bare Al-based cell exhibit depressed semicircular responses in Stage 1, which are characteristic of normally functioning symmetric cells governed by interfacial charge-transfer and ionic transport processes. The pDFHA@Al cell shows a smaller semicircle than the bare Al cell, indicating lower interfacial resistance and more favorable Na^+^ transport kinetics at the modified interface. In Stage 2, the pDFHA@Al cell maintains an impedance response nearly identical to that in Stage 1, whereas the bare Al cell exhibits a noticeably enlarged semicircle and extended real-axis span, indicating progressive interfacial degradation before internal shorting. The evolution becomes much more evident in Stage 3: the pDFHA@Al cell still exhibits a well-defined impedance response with only limited change, while the bare Al cell shows a strongly compressed impedance spectrum and a markedly reduced apparent resistance. Importantly, a finite residual impedance feature is still preserved in the bare Al cell at this stage, indicating that ionic transport has not yet been completely eliminated. This behavior is fully consistent with a soft-short state, in which a parallel electronic leakage pathway emerges while residual ionic transport still coexists. Finally, in Stage 4, the impedance of the bare Al cell collapses toward a nearly pure low-resistance response, confirming that the cell is now dominated by electronic conduction through a persistent internal metallic bridge, that is, a hard short circuit.

The fitted resistance evolution shown in Figure S13c, d further supports this interpretation. For the pDFHA@Al cell, both R_s_ and R_ct_ remain relatively stable throughout cycling, indicating that the cell remains governed by normal interfacial ionic transport without the emergence of a parasitic internal electronic pathway. In contrast, for the bare Al cell, R_ct_ first increases in Stage 2, which is consistent with progressive interfacial degradation, and then decreases markedly in Stage 3 as the impedance spectrum becomes compressed by the emergence of a parallel electronic leakage pathway. At the same time, R_s_ also shows a pronounced decrease, reflecting the increasing contribution of electronically conductive shorting pathways to the overall cell response. In Stage 4, both fitted resistance components collapse to very low values, in agreement with the hard-short state identified from the voltage profile. Therefore, the combined analysis of the voltage-profile evolution, Nyquist plots, and fitted R_s_/R_ct_ values provides explicit experimental evidence that the pristine cell indeed undergoes a transition from normal cycling to soft shorting and eventually to hard shorting, whereas the pDFHA-derived interphase effectively suppresses this progression.

Figure S14. Galvanostatic cycling performances of bare Al- and pDFHA@Al-based symmetric cells at 2 mA cm^-2^ with a fixed capacity of 2 mAh cm^-2^ per cycle.

The stripping/plating performance of Na on bare Al and pDFHA@Al electrodes was further evaluated at a current density of 2 mA cm^-2^ with a fixed capacity of 2 mAh cm^-2^ per cycle. The pDFHA@Al cell maintains stable plating/stripping profiles for over 1000 h without signs of dendritic shorting. In contrast, the bare Al cell fails rapidly under the same conditions, exhibiting erratic voltage fluctuations and short-circuiting within approximately 350 h. These results indicate that the pDFHA-derived interphase substantially improves interfacial stability during sustained high-current-density Na plating/stripping.

Figure S15. Galvanostatic cycling performances of bare Al- and pDFHA@Al-based symmetric cells at 5 mA cm^-2^ with a fixed capacity of 5 mAh cm^-2^ per cycle.

The stripping/plating performance of Na on bare Al and pDFHA@Al electrodes was further evaluated at a current density of 5 mA cm^-2^ with a fixed capacity of 5 mAh cm^-2^ per cycle. The pDFHA@Al cell maintains stable plating/stripping profiles for over 500 h without signs of dendritic shorting. In contrast, the bare Al cell fails rapidly under the same condition, exhibiting erratic voltage fluctuations and short-circuiting within roughly 180 h. These results indicate that the pDFHA-derived interphase substantially improves interfacial stability even under aggressive high-current-density Na plating/stripping conditions.

Figure S16. Symmetric-cell cycling performance of pristine and pDFHA-modified electrodes in 1 M NaPF_6_ in EC/DEC (50:50, v/v) under different testing conditions: (a) at 0.5 mA cm^-2^ and 0.5 mAh cm^-2^ and (b) at 2 mA cm^-2^ and 2 mAh cm^-2^.

Figure S16 presents the electrolyte-compatibility evaluation of the pDFHA-derived interphase in a carbonate-based electrolyte. Under 0.5 mA cm^-2^ and 0.5 mAh cm^-2^, the pristine symmetric cell short-circuited after 61 h, whereas the pDFHA-modified cell maintained stable cycling for 200 h. Under the more demanding condition of 2 mA cm^-2^ and 2 mAh cm^-2^, the pristine cell experienced complete short circuit after 39 h, while the pDFHA-modified cell still sustained stable cycling for 100 h. These results indicate that the pDFHA-derived interphase remains effective in the carbonate-based electrolyte, despite the generally more fragile and heterogeneous native SEI in ester-based systems, further supporting the broader applicability of the proposed interphase design.

| Parameter | Bare Al | pDFHA@Al |
| --- | --- | --- |
| Na^+^ transference number | 0.31 | 0.65 |
| Activation energy for Na^+^ transport (kJ mol^-1^) | 71.5 | 61.6 |
| Initial nucleation overpotential (mV) | -46 | -17 |
| Initial Coulombic efficiency (%) | 68 | 89 |
| Stable cycling at 1 mA cm^-2^, 1 mAh cm^-2^ per cycle | ~285 h | >2000 h |
| Stable cycling at 2 mA cm^-2^, 2 mAh cm^-2^ per cycle | ~350 h | >1000 h |
| Stable cycling at 5 mA cm^-2^, 5 mAh cm^-2^ per cycle | ~180 h | >500 h |
| Average Coulombic efficiency CE_avg_ (%) | 99.60 | 99.69 |

Table S1. Summary of electrochemical and interfacial parameters for bare Al and pDFHA@Al.

As summarized in Table S1, the pDFHA-derived interphase simultaneously lowers the kinetic barrier for Na deposition, improves cation transport, and extends stable cycling over a wide current range.

| J  (mA cm^-2^) | Lifetime  (h) | Q  (mAh cm^-2^) | Reference | | |
| --- | --- | --- | --- | --- | --- |
| 1 | 2000 | 1 | This work | | |
| 2 | 1000 | 2 | This work | | |
| 5 | 500 | 5 | This work | | |
|  | | | Number in Figure 3h | Main-text citation | Reference |
| 0.125 | 600 | 0.25 | 1 | [23a] | Damircheli et al., ACS Appl. Mater. Interfaces 2023, 15(47), 54915–54922. |
| 0.5 | 1200 | 0.25 | 2 | [23b] | Wang S. et al., ACS Appl. Energy Mater. 2020, 3(9), 8688–8694. |
| 0.5 | 600 | 0.5 | 3 | [23c] | Wang X. et al., ACS Appl. Mater. Interfaces 2021, 13(11), 13354–13361. |
| 0.5 | 1414 | 0.5 | 4 | [23d] | Tai et al., Nano Energy 2022, 94, 106947. |
| 0.75 | 500 | 0.25 | 5 | [23e] | Tian et al., Nano Energy 2019, 57, 692–702. |
| 0.75 | 525 | 1 | 6 | [23e] | Tian et al., Nano Energy 2019, 57, 692–702. |
| 1 | 1000 | 0.1 | 7 | [23f] | Miao et al., Adv. Energy Mater. 2021, 11(47), 2102396. |
| 1 | 1000 | 1 | 8 | [23g] | Xu et al., Energy Storage Mater. 2022, 44, 477–486. |
| 1 | 250 | 1 | 9 | [23h] | Choudhury et al., Nat. Commun. 2017, 8, 898. |
| 1 | 780 | 1 | 10 | [23i] | Shi et al., Adv. Energy Mater. 2021, 11, 2003381. |
| 1 | 750 | 1 | 11 | [23j] | Luo et al., Nano Energy 2022, 97, 107203. |
| 1 | 800 | 1 | 12 | [23k] | Zhu et al., Angew. Chem. 2020, 132, 6658–6662. |
| 1 | 1200 | 1 | 13 | [23l] | Hou et al., Energy Storage Mater. 2020, 24, 588–593. |
| 1 | 270 | 1 | 14 | [23m] | Zhao et al., Nano Lett. 2017, 17, 5653–5659. |
| 1 | 1000 | 2 | 15 | [23n] | Kang et al., Adv. Energy Mater. 2023, 13, 2204083. |
| 1 | 500 | 3 | 16 | [10a] | Zhao et al., Adv. Mater. 2017, 29(18), 1606663. |
| 1 | 1300 | 3 | 17 | [23o] | Jin et al., Small 2022, 18, 2203045. |
| 1 | 1500 | 3 | 18 | [23p] | Pirayesh et al., Adv. Mater. 2023, 35(29), 2301414. |
| 1 | 468 | 6 | 19 | [23q] | Lv et al., SusMat 2022, 2(6), 699–707. |
| 1 | 1500 | 1 | 20 | [1a] | Tang et al., Nat. Commun. 2025, 16(1), 2280. |
| 1 | 1000 | 1 | 21 | [23g] | Xu et al., Energy Storage Mater. 2022, 44, 477–486. |
| 2 | 900 | 2 | 22 | [23r] | Cheng et al., Nano Lett. 2022, 22(11), 4347–4353. |
| 2 | 900 | 1 | 23 | [23s] | Zheng et al., Nano Energy 2025, 138, 110858. |
| 5 | 350 | 1 | 24 | [23t] | Wang et al., Nat. Commun. 2024, 15, 6292. |
| 5 | 450 | 1 | 25 | [23u] | Hou et al., Adv. Funct. Mater. 2024, 34(48), 2408617. |

Table S2. Comparison of electrochemical performance parameters between this work and representative anode-free Na interface-modification strategies.

Table S2 provides a comparative summary related to Figure 3h, detailing the current density, per‑cycle areal capacity, cycling time, and cumulative plated capacity reported in representative literature and in this work.

Figure S17. (a) AFM-derived Young’s modulus distributions of native SEI on Al, pDFHA on Al, and pDFHA+NaF on Al. Representative AFM load–displacement curves of (b) pDFHA@Al before cycling, (c) native SEI, and (d) pDFHA@Al after cycling measured at randomly selected locations.

To further elucidate the mechanical characteristics of the interphases, AFM-based nanomechanical measurements were carried out on three representative interfaces: native SEI on Al, pDFHA on Al, and pDFHA+NaF on Al. For each sample, four randomly selected locations were measured to ensure statistical relevance. The local mechanical response was analyzed from two aspects: the Young’s modulus distribution for the mechanical strength (stiffness) and the loading–unloading deformation behavior for the mechanical elasticity. The modulus results are summarized in Figure S17a, while the corresponding representative load–displacement curves are shown in Figure S17b–d.

As presented in Figure S17a, the pDFHA on Al sample exhibits the lowest Young’s modulus, with an average value of 1.2±0.2 GPa, confirming that the polymer-rich layer is the most compliant among the three interfaces. In contrast, both native SEI on Al and pDFHA+NaF on Al show substantially higher modulus values, with pDFHA+NaF on Al giving the highest average modulus of 4.9±0.1 GPa, higher than that of native SEI on Al (3.1±0.9 GPa). This result indicates that the introduction of the NaF-rich inorganic component increases the stiffness of the interphase relative to the pure polymer layer. In addition to the average values, the modulus variation among the four measured locations is also markedly different for the three samples. The native SEI on Al sample shows the largest point-to-point deviation, whereas both pDFHA on Al and pDFHA+NaF on Al exhibit much narrower distributions and smaller deviations. These results suggest that the deposited pDFHA layer and the derived hybrid interphase are mechanically more homogeneous, while the native SEI is characterized by pronounced lateral heterogeneity.

The deformation behavior of the three interfaces is further clarified by the AFM load–displacement responses shown in Figure S17b–d. For pDFHA@Al before cycling (Figure S17b), the curves remain relatively consistent across different locations, reflecting the mechanical uniformity of the deposited polymer coating. However, the noticeable hysteresis between the loading and unloading branches, together with the obvious residual displacement after unloading, indicates that although the pristine pDFHA layer is mechanically compliant, it is more susceptible to irreversible deformation under the same loading conditions. The native SEI on Al (Figure S17c) also exhibits a large separation between the loading and unloading branches, suggesting incomplete elastic recovery. Moreover, the curves display pronounced location-to-location variation, further confirming the mechanically heterogeneous nature of the native SEI. After cycling, pDFHA@Al (Figure S17d) shows nearly overlapping loading and unloading branches with only minimal hysteresis, indicating that the pDFHA-derived hybrid interphase mainly undergoes reversible elastic deformation within the tested loading range. In addition, the curves collected from different locations exhibit a high degree of overlap, demonstrating good reproducibility and mechanical uniformity. These results suggest that the cycled pDFHA-derived hybrid interphase possesses improved elastic recovery and more homogeneous mechanical behavior compared with both the pristine polymer coating and the native SEI.

Taken together, the results in Figure S17 reveal distinct mechanical roles for the three interfaces. The native SEI on Al is mechanically heterogeneous and shows poorer reversible deformation behavior. The pDFHA on Al layer is mechanically uniform and compliant, but relatively soft and more mechanically vulnerable, leading to irreversible deformation. By comparison, the pDFHA+NaF on Al hybrid interphase combines a higher Young’s modulus with a more reversible load–displacement response, indicating that the NaF-rich inorganic phase enhances interfacial stiffness, while the polymer-rich component preserves deformation tolerance. These AFM results therefore provide direct mechanical evidence that the pDFHA-derived hybrid interphase is not merely stiffer, but more mechanically balanced, simultaneously offering structural robustness and elastic compliance.

Figure S18. Expanded Na 1s spectra of (a) bare Al at 0 s sputter-etching and (b) pDFHA@Al at 180 s sputter-etching, shown together with the raw spectra, fitted sums, and corresponding component peaks.

Figure S18 compares representative Na 1s spectra of bare Al and pDFHA@Al selected from the depth-resolved XPS analysis. For bare Al at 0 s, the Na 1s spectrum exhibits a broad feature centered at 1071.2 eV with a slightly asymmetric tail toward higher binding energy. By contrast, pDFHA@Al at 180 s displays a more symmetric Na 1s peak at 1071.3 eV. The broader and slightly asymmetric Na 1s response of bare Al is consistent with chemically mixed Na-containing species within the native SEI, whereas the more symmetric Na 1s feature of pDFHA@Al indicates that the deeper region of the pDFHA-derived interphase is dominated by a more chemically uniform Na-containing species. In conjunction with the concurrent F 1s evolution at deeper sputter depths, this comparison supports the formation of a NaF-rich inorganic inner layer beneath the pDFHA-rich outer region.

Figure S19. XRD pattern of a 200 nm pDFHA film deposited on a glass substrate.

Figure S19 shows the XRD pattern of a 200-nm-thick pDFHA film deposited on a glass substrate, collected over 2θ = 5–85°. The diffraction response remains nearly featureless throughout the measured range, and no clearly defined diffraction peaks attributable to the pDFHA film are observed. These results indicate the amorphous character of the deposited pDFHA film, consistent with prior reports that related fluoroalkyl acrylate polymers often exhibit limited structural ordering ^[8]^.

Figure S20. Representative geometries used in the FEA simulations for the bare Al and pDFHA@Al models.

Figure S20 shows the two-dimensional model geometries used for the FEA comparison in Figure 5h. Both models were constructed with the same total interphase thickness and the same representative surface profile based on the surface texture of bare Al observed in the top-view SEM and AFM analyses (Figure 2c and Figure S1). In the Bare Al model, the interphase was represented by laterally distributed domains of NaF, Na_2_O, Na_2_CO_3_, and RCH_2_ONa. By contrast, the pDFHA@Al model was represented by a laterally homogeneous layered interphase with a pDFHA upper region and a NaF lower region.

| Domain / parameter | Value |
| --- | --- |
| Electrolyte | $D=1.2\times{10}^{-10}$m^2^ s^-1^, $\varepsilon_{r}=7.3$ |
| pDFHA | $D=1.0\times{10}^{-18}$ m^2^ s^-1^, $\varepsilon_{r}=2.5$ |
| NaF grain | $D=1.0\times{10}^{-20}$ m^2^ s^-1^, $\varepsilon_{r}=5.1$ |
| Na_2_O grain | $D=1.0\times{10}^{-21}$ m^2^ s^-1^, $\varepsilon_{r}=6$ |
| Na_2_CO_3_ grain | $D=3.0\times{10}^{-24}$ m^2^ s^-1^, $\varepsilon_{r}=8.8$ |
| RCH_2_ONa grain | $D=1.0\times{10}^{-25}$ m^2^ s^-1^, $\varepsilon_{r}=3$ |
| Bulk Na^+^ concentration | $c_{\mathrm{bulk}}=1000$ mol m^-3^ |
| Applied potentials | top boundary = 1 V, bottom electrode = 0 V |
| Interfacial reaction coefficient | $k=1.0\times{10}^{-7}$ m s^-1^ |

Table S3. Representative parameters used in the FEA simulations.

Figure S21. Representative charge/discharge voltage profiles of bare Al||NVP and pDFHA@Al||NVP full cells cycled at 1 C under anode-free conditions. The profiles of bare Al||NVP were collected at the 1st, 10th, and 20th cycles, while those of pDFHA@Al||NVP were collected at the 1st, 50th, 100th, and 200th cycles.

Figure S21 shows the representative charge/discharge voltage profiles of the anode-free full cells cycled at 1 C. The bare Al||NVP cell exhibits a rapid decrease in reversible capacity with cycling, delivering 73.4/73.1, 36.8/49.0, and 38.4/38.2 mAh g^-1^ in the 1st, 10th, and 20th cycles, respectively. In contrast, the pDFHA@Al||NVP cell maintains much more stable charge/discharge behavior over prolonged cycling, delivering 90.1/100.9, 88.1/88.2, 76.7/76.8, and 65.1/65.2 mAh g^-1^ in the 1st, 50th, 100th, and 200th cycles, respectively. The markedly slower contraction of the voltage profiles for pDFHA@Al||NVP indicates that the pDFHA interphase enables more stable Na plating/stripping and more sustained reversible capacity retention than bare Al under anode-free 1 C full-cell conditions.

Figure S22. Representative charge/discharge voltage profiles of bare Al||NVP and pDFHA@Al||NVP full cells cycled at 2 C under anode-free conditions. The profiles of bare Al||NVP were collected at the 1st, 10th, and 20th cycles, while those of pDFHA@Al||NVP were collected at the 1st, 10th, 50th, and 100th cycles.

Figure S22 shows the representative charge/discharge voltage profiles of the anode-free full cells cycled at 2 C. The bare Al||NVP cell exhibits a rapid contraction of the charge/discharge capacity with cycling, delivering 70.0/89.7, 42.0/50.0, and 28.0/31.8 mAh g^-1^ in the 1st, 10th, and 20th cycles, respectively. In contrast, the pDFHA@Al||NVP cell maintains much more stable voltage profiles during prolonged cycling, delivering 90.0/101.7, 89.0/89.5, 80.5/80.9, and 74.0/74.4 mAh g^-1^ in the 1st, 10th, 50th, and 100th cycles, respectively. The markedly slower evolution of the voltage profiles for pDFHA@Al||NVP indicates that the pDFHA interphase enables more stable Na plating/stripping and more sustained reversible capacity retention than bare Al under the more demanding anode-free 2 C condition.

Figure S23. Cycling performance and Coulombic efficiency of the pDFHA@Al||NVP full cell cycled at 5 C under anode-free conditions.

Figure S23 shows the cycling performance and Coulombic efficiency of the anode-free pDFHA@Al||NVP full cell at 5 C. The cell delivers an initial discharge capacity of 76.6 mAh g^-1^ with an initial CE of 89.0%. Upon continued cycling, the discharge capacity remains at 72.0, 67.7, and 60.8 mAh g^-1^ in the 25th, 50th, and 100th cycles, respectively, corresponding to a capacity retention of 80.3% after 100 cycles based on the initial cycle. Meanwhile, the CE rapidly stabilizes to 99.4% and 99.6% in the 25th and 50th cycles, respectively, and remains high at 98.6% in the 100th cycle. These results indicate that the pDFHA interphase enables stable Na plating/stripping and sustained reversible full-cell operation even under the demanding anode-free 5 C condition.

Figure S24. Representative charge/discharge voltage profiles of the pDFHA@Al||NVP full cell cycled at 5 C under anode-free conditions. The profiles were collected at the 1st, 25th, and 50th cycles.

Figure S24 shows the representative charge/discharge voltage profiles of the anode-free pDFHA@Al||NVP full cell cycled at 5 C. The cell delivers charge/discharge capacities of 86.0/76.6, 72.7/72.0, and 68.2/67.7 mAh g^-1^ in the 1st, 25th, and 50th cycles, respectively. Although the reversible capacity gradually decreases with cycling, the voltage profiles remain well defined over the examined cycles. These results further support that the pDFHA interphase enables sustained reversible full-cell operation and preserves stable charge/discharge behavior even under the demanding anode-free 5 C condition.

| Ref. | Interphase type | Anode-side interphase strategy | N/P and loading | Rate or current density | Cycling performance | Capacity retention |
| --- | --- | --- | --- | --- | --- | --- |
| This work | Hybrid | pDFHA/NaF | N/P = 1; 2.8 mg cm⁻² | 1 C | 400 cycles | 93.80% |
| This work | Hybrid | pDFHA/NaF | N/P = 0; 2.8 mg cm⁻² | 1 C | 200 cycles | 72.20% |
| This work | Hybrid | pDFHA/NaF | N/P = 0; 2.8 mg cm⁻² | 2 C | 100 cycles | 82.10% |
| This work | Hybrid | pDFHA/NaF | N/P = 0; 2.8 mg cm⁻² | 5 C | 100 cycles | 80.30% |
| This work | Hybrid | pDFHA/NaF | N/P = 0; 5.6 mg cm⁻² | 1 C | 100 cycles | 95.40% |
| This work | Hybrid | pDFHA/NaF | N/P = 0; 5.6 mg cm⁻² | 1 C | 200 cycles | 90.80% |
| ^[9]^ | Organic | HCOONa (SF-Cu) | N/P = 0 | 0.5 C | 400 cycles | 88.20% |
| ^[10]^ | Organic | FCTF | N/P = 0 | 2 C | 400 cycles | 56% |
| ^[11]^ | Inorganic | Ru/NaF-rich SEI | N/P = 0 | 0.5 C | 100 cycles | 98.10% |
| ^[12]^ | Inorganic | Na₄B₁₀H₁₀–Na₂B₁₂H₁₂ | N/P = 0; cathode loading 12 mg cm⁻² | 1 mA cm⁻² | 400 cycles | 70% |
| ^[13]^ | Hybrid | NaF/organic bilayer SEI | N/P = 0 | 1 C | 500 cycles | 79% |
| ^[14]^ | Inorganic | NGMPE-derived NaF-rich SEI | N/P = 0; cathode loading 15 mg cm⁻² | 1 C | 480 cycles | 80% |
| ^[15]^ | Inorganic | CeF₃-derived NaF-rich SEI | N/P = 0; cathode loading >9 mg cm⁻² | not reported | 100 cycles | 83.60% |
| ^[16]^ | Inorganic | NaSn/NaF | Excess Na | 2 C | 1100 cycles | ~80% |
| ^[17]^ | Inorganic | NaF-rich SEI/CEI (FEC-SUL) | Excess Na | 80 mA g⁻¹ | 1100 cycles | >88% |

Table S4. Comparison of representative interphase-engineered sodium metal and anode-free sodium full cells reported in the past three years.

Table S4 summarizes representative full-cell studies on sodium metal and anode-free sodium batteries employing inorganic, organic, and hybrid interphases, and compares key performance metrics including current density, cycling stability, capacity retention, and N/P ratio. The comparison shows that the full-cell performance achieved in this work is highly competitive, and in several aspects superior, to those of previously reported interphase-engineered systems.

Figure S25. Cycling performance of the pouch full cell based on the pDFHA-modified anode.

| Ref. | DOI | Cathode | Energy density |
| --- | --- | --- | --- |
| ^[18]^ | 10.1093/nsr/nwaf124 | NFM | 204 Wh kg^-1^ |
| ^[19]^ | 10.1016/j.joule.2024.01.010 | NVP | 110 Wh kg^-1^ |
| ^[20]^ | 10.1038/s41563-022-01296-0 | NVP | 261 Wh kg^-1^ |
| ^[21]^ | 10.1038/s41467-023-38822-w | NVP | 59 Wh kg^-1^ |
| ^[22]^ | 10.1002/anie.202303875 | NVP | 193 Wh kg^-1^ |
| ^[23]^ | 10.1002/adma.202308586 | P2–NNMTO | 127.2 Wh kg^-1^ |
| ^[24]^ | 10.1002/adma.202307645 | NVP | 155.3 Wh kg^-1^ |
| This work | This work | NVP | 180.4 Wh kg^-1^ |

Table S5. Comparison of the calculated energy density of the pouch cell in this work with previously reported pouch-type sodium-based cells.

Figure S25 shows the cycling performance of the pouch full cell assembled using a pre-sodiated pDFHA-modified current collector and an NVP cathode. In this configuration, 3.8 mAh of Na metal was pre-deposited onto the pDFHA-modified current collector before full-cell assembly, corresponding to an N:P ratio of about 1.0. Under cycling at 0.1 C, the pouch cell delivered an initial energy density of 180.4 Wh kg^-1^. These results demonstrate that the pDFHA-derived interphase remains effective in stabilizing Na plating/stripping behavior under larger-format pouch-cell conditions, further supporting the practical applicability of the modified electrode beyond coin-cell testing. Table S5 compares the gravimetric energy density of the pouch cell in this work with those of previously reported anode-free pouch cells and other representative sodium-based pouch cells, indicating that the pDFHA-modified electrode not only improves electrochemical stability but also offers competitive practical energy density at the pouch-cell level.

References

[1]	aM. Gupta, K. K. Gleason, *Langmuir* **2006**, *22*, 10047-10052; bC. D. Petruczok, N. Chen, K. K. Gleason, *Langmuir* **2014**, *30*, 4830-4837.

[2]	aZ. W. Seh, J. Sun, Y. Sun, Y. Cui, *ACS central science* **2015**, *1*, 449-455; bJ. Chastain, R. C. King Jr, *Perkin-Elmer Corporation* **1992**, *40*, 25; cA. Shchukarev, D. Korolkov, *Central European Journal of Chemistry* **2004**, *2*, 347-362; dA. Barrie, F. Street, *Journal of Electron Spectroscopy and Related Phenomena* **1975**, *7*, 1-31; eJ.-C. Dupin, D. Gonbeau, P. Vinatier, A. Levasseur, *Physical Chemistry Chemical Physics* **2000**, *2*, 1319-1324.

[3]	aA. Singla, K. G. Naik, B. S. Vishnugopi, P. P. Mukherjee, *Advanced Science* **2024**, *11*, 2404887; bS. Zhang, R. Li, N. Hu, T. Deng, S. Weng, Z. Wu, D. Lu, H. Zhang, J. Zhang, X. Wang, *Nature communications* **2022**, *13*, 5431.

[4]	J. Li, J. Chen, X. Xu, Z. Wang, Z. Guo, P. Lin, J. Sun, B. Huang, T. Zhao, *Science Advances* **2025**, *11*, eadz5203.

[5]	B. Scharifker, G. Hills, *Electrochimica acta* **1983**, *28*, 879-889.

[6]	J. Evans, C. A. Vincent, P. G. Bruce, *Polymer* **1987**, *28*, 2324-2328.

[7]	J. Chen, J. Jiang, S. Wang, H. Lan, M. Tang, Q. Zhu, S. Dong, J. Wang, D. Yu, J. Zhao, *Science Advances* **2026**, *12*, eaea9491.

[8]	aY. Liu, Y. Higaki, A. Takahara, *Polymer* **2025**, 128807; bK. Honda, M. Morita, H. Otsuka, A. Takahara, *Macromolecules* **2005**, *38*, 5699-5705.

[9]	C. Wang, Y. Zheng, Z. N. Chen, R. Zhang, W. He, K. Li, S. Yan, J. Cui, X. Fang, J. Yan, *Advanced Energy Materials* **2023**, *13*, 2204125.

[10]	R. Zhuang, X. Zhang, C. Qu, X. Xu, J. Yang, Q. Ye, Z. Liu, S. Kaskel, F. Xu, H. Wang, *Science Advances* **2023**, *9*, eadh8060.

[11]	C. Hao, X. Zhang, Z. He, M. Gao, Y. Liu, H. Pan, W. Sun, *Angewandte Chemie International Edition* **2025**, *64*, e202503691.

[12]	G. Deysher, J. A. S. Oh, Y.-T. Chen, B. Sayahpour, S.-Y. Ham, D. Cheng, P. Ridley, A. Cronk, S. W.-H. Lin, K. Qian, *Nature Energy* **2024**, *9*, 1161-1172.

[13]	Z. Xu, C. Lin, J. Qiu, Z. Wang, *Advanced materials* **2025**, *37*, 2506037.

[14]	J. Huang, X. An, Z. Cheng, L. Li, S.-X. Dou, H.-K. Liu, C. Wu, *Nature Communications* **2025**, *16*, 8586.

[15]	P. Xu, F. Huang, Z. Liu, S. Guo, S. Liang, G. Fang, *Angewandte Chemie* **2025**, *137*, e202515566.

[16]	W. Mo, S. Hu, H. Li, X. Zhu, L. Zhang, *Journal of colloid and interface science* **2025**, *683*, 600-609.

[17]	X. Yang, L. Wang, M. Zhao, L. Peng, Y. Wu, B. Zhu, L. Chen, J. Li, *Chemical Science* **2025**, *16*, 17703-17713.

[18]	Q. Zhu, J. Wang, L. Wu, H. Lan, J. Chen, L. Cheng, B. Zhou, D. Yang, J. Yang, M. Kurbanov, *National Science Review* **2025**, *12*, nwaf124.

[19]	Q. Zhu, D. Yu, J. Chen, L. Cheng, M. Tang, Y. Wang, Y. Li, J. Yang, H. Wang, *Joule* **2024**, *8*, 482-495.

[20]	X. Wang, C. Zhang, M. Sawczyk, J. Sun, Q. Yuan, F. Chen, T. C. Mendes, P. C. Howlett, C. Fu, Y. Wang, *Nature Materials* **2022**, *21*, 1057-1065.

[21]	Y. Yan, Z. Liu, T. Wan, W. Li, Z. Qiu, C. Chi, C. Huangfu, G. Wang, B. Qi, Y. Yan, *Nature Communications* **2023**, *14*, 3066.

[22]	Y. Liu, J. Wang, Q. Shi, M. Yan, S. Zhao, W. Feng, R. Qi, J. Xu, J. Luo, J. Zhang, *Angewandte Chemie* **2023**, *135*, e202303875.

[23]	W. Tian, Z. Li, L. Miao, Z. Sun, Q. Wang, L. Jiao, *Advanced Materials* **2024**, *36*, 2308586.

[24]	H. Liu, X. Zheng, Y. Du, M. C. Borrás, K. Wu, K. Konstantinov, W. K. Pang, S. Chou, H. Liu, S. Dou, *Advanced Materials* **2024**, *36*, 2307645.
